# Targeting Regulatory Noncoding RNAs in Human Cancer: The State of the Art in Clinical Trials

**DOI:** 10.3390/pharmaceutics17040471

**Published:** 2025-04-04

**Authors:** Roberto Piergentili, Stefano Sechi

**Affiliations:** Istituto di Biologia e Patologia Molecolari del Consiglio Nazionale delle Ricerche, Dipartimento di Biologia e Biotecnologie, Università Sapienza di Roma, Piazzale Aldo Moro 5, 00185 Rome, Italy; roberto.piergentili@cnr.it

**Keywords:** noncoding RNA, clinical trial, cancer, biomarker, oncological therapy

## Abstract

Noncoding RNAs (ncRNAs) are a heterogeneous group of RNA molecules whose classification is mainly based on arbitrary criteria such as the molecule length, secondary structures, and cellular functions. A large fraction of these ncRNAs play a regulatory role regarding messenger RNAs (mRNAs) or other ncRNAs, creating an intracellular network of cross-interactions that allow the fine and complex regulation of gene expression. Altering the balance between these interactions may be sufficient to cause a transition from health to disease and vice versa. This leads to the possibility of intervening in these mechanisms to re-establish health in patients. The regulatory role of ncRNAs is associated with all cancer hallmarks, such as proliferation, apoptosis, invasion, metastasis, and genomic instability. Based on the function performed in carcinogenesis, ncRNAs may behave either as oncogenes or tumor suppressors. However, this distinction is not rigid; some ncRNAs can fall into both classes depending on the tissue considered or the target molecule. Furthermore, some of them are also involved in regulating the response to traditional cancer-therapeutic approaches. In general, the regulation of molecular mechanisms by ncRNAs is very complex and still largely unclear, but it has enormous potential both for the development of new therapies, especially in cases where traditional methods fail, and for their use as novel and more efficient biomarkers. Overall, this review will provide a brief overview of ncRNAs in human cancer biology, with a specific focus on describing the most recent ongoing clinical trials (CT) in which ncRNAs have been tested for their potential as therapeutic agents or evaluated as biomarkers.

## 1. Introduction

Cancer has a significant impact, not only on the physical and mental health of the person affected but also on society, considering the significant economic impact due to the high costs of the development and use of effective therapies. Furthermore, according to the World Health Organization (WHO) (see Table 1 for this and other abbreviations used throughout the text), cancer resulted in almost 10 million deaths in 2020, making it one of the leading causes of death worldwide [1].

Over time, the definition of cancer has changed several times in relation to new discoveries, especially in the field of molecular biology and genetics, and more generally with technical–scientific advancements. Many works use definitions that are largely consistent with that provided by the NCI: “Cancer is a disease in which some of the body’s cells grow uncontrollably and spread to other parts of the body” [2]. However, this definition does not consider the genetic and molecular aspects that lead to the malignant transformation of the cell or how these aspects vary over time. Consequently, this definition deeply simplifies the scenario that leads to carcinogenesis, the understanding of which is of fundamental importance for its diagnosis, prognosis, and therapy.

Each of the approximately 36 trillion cells that constitute the adult male body, and approximately 28 trillion in the female one [3], can potentially develop a tumor, which may have a solid or liquid structure. Solid tumors form an abnormal mass (or lumps) in specific organs or tissues, whereas liquid tumors do not form a solid mass, and cancer cells can circulate through either the bloodstream or lymphatic system [4,5,6].

The NCI lists more than 150 different types of tumors organized by organ location [7], but this number significantly increases considering that many tumors can be further divided into distinct subtypes based on their different “mutational signatures”. The incidence of these tumors is highly variable. In fact, while some tumors are particularly widespread in the population, such as BrC or PrC (1–3%), others, such as stomach cancer and LarC, occur in less than 15/100,000 people/year and are considered rare by the NCI [8].

Cancer-causing mutations are irreversible cellular mutations that affect DNA, both nDNA and mtDNA, and occur in regions that are necessary for proper cellular function and health. Cancer-causing mutations are known as driver mutations, and the affected DNA regions can be PC or nPC regions of the genome [9,10,11,12]. Initially, cancer-causing mutations induce changes in one or a few cells through a multistep process. These changes accumulate over time, arise from independent events, and undergo selection. These mutations allow a healthy cell to acquire new functional capabilities, collectively described as hallmarks of cancer, leading to its neoplastic transformation [13].

Furthermore, mutations continue to accumulate during subsequent cell divisions, producing heterogeneous progeny hosting different genetic profiles. This aspect implies that the tumor is composed of multiple subclones that share a common ancestor. These subclones can further diverge and expand simultaneously over time, acquiring different characteristics, such as increased fitness or intratumor variability, i.e., the simultaneous coexistence of genetically, molecularly, and phenotypically distinct cell populations [14]. Comprehensive reviews regarding this topic are available in the literature [15,16,17].

PC regions are sequences contained in genes that are translated into proteins. In particular, PCs represent only about 1–2% of the human genome, the remainder of which, about 98%, is composed of nPC regions [18]. This aspect is particularly important because an analysis of the SNVs identified in GWAS shows that more than 88% of disease- and trait-associated variants fall within nPC regions of the genome [19,20], showing that mutations causing protein alteration and dysfunction represent only a minority of the possible causes of tumorigenesis.

NPC regions are highly heterogeneous DNA sequences, both in terms of function and genomic localization, and participate in every biological process. Some nPC sequences have structural functions, such as the telomere and the centromere, which are both composed mainly of satellite DNA (high frequency of repetitive DNA sequences) and are considered essential both for the stability of the genome and for the correct carrying out of cell division [21,22]. Other nPC regions have a regulatory function and can be interspersed in the genome as transposons, enhancers, silencers, and insulators or form part of the canonical structure of a gene, such as the gene promoter and mRNA UTR. Finally, there are some types of nPCs that can be transcribed into RNA molecules, which in turn divide into constitutive or regulatory RNAs.

The distinction between the various types of nPC is unclear, and, often, the same region can be involved in multiple pathways. For example, TE are repeated and interspersed sequences capable of moving from one position to another in the genome of the same cell. Transposons, through their movement, produce genetic diversity mainly by a phenomenon called exon shuffling. Indeed, when the excision is not perfect, a TE can carry with it genomic sequences, and, if the juxtaposition of two previously unrelated exons occurs, potentially new gene products can be created. TE insertion can also cause damage if it occurs within a sequence that becomes nonfunctional or abnormally regulated with respect to cellular needs [23,24]. TEs are involved in the biogenesis of some ncRNAs with regulatory functions, such as miRNAs or piRNAs [25,26]. The processing of some constitutive RNAs can also generate regulatory ncRNAs [27,28,29,30,31,32].

The two main types of nPC regions with regulatory functions localized within the structure of a gene are the promoter, which plays a role in regulating the rate of transcription initiation of the gene, and the UTR, with sequences present at both the 3′ and 5′ ends of the gene and playing crucial roles in the post-transcriptional regulation of gene expression. The gene may also contain another type of nPC represented by introns, which are nucleotide sequences that separate two contiguous exons and from which, in some cases, the ncRNA could originate. Some mutations in the constituent elements of genes (promoters, UTRs, introns, and exons) could alter the levels of gene expression or lead to the synthesis of a nonfunctional protein product. If these mutations contribute to promoting carcinogenesis, they are called driver mutations, and the affected genes are called driver genes [33]. Cancer driver genes are broadly divided into two functional classes: oncogenes and tumor suppressor genes [34]. Oncogenes are usually involved in controlling cell proliferation and division, and, through gain-of-function mutations, they increase their activity compared to normal conditions. Meanwhile, tumor suppressor genes usually inhibit cell growth and division, promote DNA repair, and activate cell cycle checkpoints. The inactivation of tumor suppressors by loss-of-function mutations eliminates regulatory control over their targets, thus promoting the development of carcinogenesis.

### Constitutive and Regulatory ncRNAs

**Figure 1:**
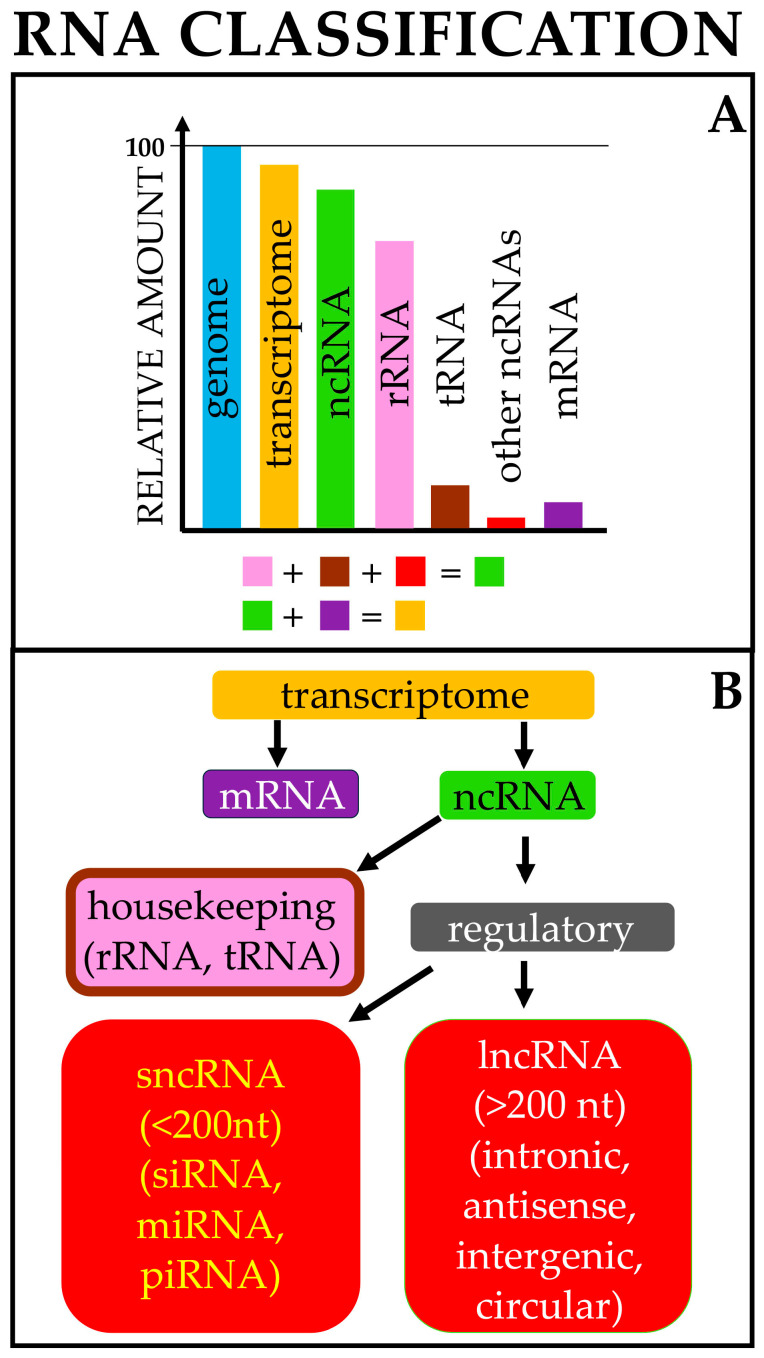
RNA origin and classification. (**A**) Approximately 95% of the genome is transcribed (transcriptome). Of this, around 90–95% is composed of noncoding RNAs (ncRNAs), mostly rRNAs and tRNAs; the remainder consists of mRNAs. (**B**) Broad classification of cellular RNAs; for each class, only representative RNAs are reported. The list is not intended to be comprehensive. Color codes are the same in both figure parts.

Finally, other types of nPC sequences are transcribed into RNA molecules. Interestingly, approximately 75–80% of the human genome is transcribed into RNA, indicating that it transcribes tens of thousands of RNA molecules. These RNA molecules are collectively called ncRNAs [35,36,37] and can be divided into constitutive and regulatory ncRNAs (Figure 1) on the basis of their main function, with the former being abundantly and ubiquitously expressed in all cell types and providing essential functions to the organism, such as transcription or translation, and the latter being involved in the regulation of target gene expression.

Constitutive RNAs include tRNAs, rRNAs, snRNAs, snoRNAs, and TERCs. The function of tRNAs is to carry a specific amino acid, which, during translation, is added to the nascent protein chain [38]. Ribosomal RNAs fold to form secondary structures and play a structural and functional role within ribosomes, thus contributing to the enzymatic activity of the ribosome complex that is required for protein synthesis. There are four rRNA molecules in the ribosome that differ in sequence length and sedimentation coefficient: 5.8 S (156 nt), 18 S (1869 nt), 28 S (5070 nt), and 5 S (121 nt) [39,40]. snRNAs are involved in the formation of the spliceosome, a complex that allows the correct excision of introns (splicing) from the pre-mRNA sequence. There are many types of spliceosomes, distinguished by the combination of proteins and snRNAs used for the activity of the complex. There are five snRNAs and they have an approximate length of 100–200 nt: U1, U2, U4, U5, and U6 [41]. snoRNAs have a sequence length between 60 and 300 nt, are widely present in the nucleoli of eukaryotic cells, and are mainly encoded in the intron region of the gene transcribed by RNA polymerase II [42]. snoRNAs guide chemical modifications of other RNAs, such as rRNAs, tRNAs, snRNAs, and some types of mRNAs [43,44]. The modifications imposed by snoRNAs affect the stability and folding of RNAs, as in the case of rRNAs and tRNAs. In addition, several studies suggest that some snoRNAs can play miRNA-like roles because they are involved in the regulation of gene expression through the regulation of alternative splicing or the inhibition of the mRNAs of target genes [45,46].

Regulatory ncRNAs, which include miRNAs, siRNAs, piRNAs, lncRNAs, and circRNAs, will be discussed in detail in the following paragraphs.

The described genomic elements that constitute both the protein-coding and non-protein-coding regions are dependent on each other, implying that mutations affecting one region can affect the other and vice versa. For example, DNA methylation is an epigenetic mechanism that involves the transfer of a methyl group by specific enzymes, namely DNMTs, and accessory proteins on CpG islands. A CpG island refers to an area in the genome with a higher frequency of short stretches of palindromic DNA, in which a cytosine nucleotide is followed by a guanine nucleotide (with “p” indicating the phosphate bond between them). The methylation of CpG islands, usually found in promoters, allows them to regulate gene expression by recruiting proteins involved in gene repression or by inhibiting the binding of transcription factors to DNA. However, DNA methylation can also influence the expression of regulatory RNAs, including lncRNAs [47] and miRNAs [48,49]. In particular, miRNAs are small, ss ncRNAs that, through various mechanisms (described in Section 4.2), are involved in the regulation of gene expression. Aberrant DNA methylation can lead to either the upregulation or downregulation of miRNA expression, which can be associated with tumorigenesis [50,51]. Conversely, miRNAs can regulate DNA methylation in two ways: by modulating DNMTs’ activity [52,53] or by modulating the functions of accessory proteins that play a role in DNA methylation [54,55].

The existing link between PC and nPC regions suggests a plethora of very complex mechanisms by which carcinogenesis originates, which are very difficult to elucidate due to changes over time. Therefore, understanding how tumor evolution influences disease progression and how these processes are influenced by environmental factors and therapeutic treatments remains fundamental to develop not only new diagnostic and prognostic markers but also new therapeutic approaches.

For many decades, cancer patients have relied on three main different therapeutic approaches that can be used individually or in combination with each other: the surgical removal of the tumor mass, radiotherapy, and chemotherapy. These approaches have had an overall positive effect on morbidity and mortality in many types of cancer, despite the observation that the response to treatment can greatly vary from patient to patient, as it is influenced by parameters such as the cancer type and stage.

Over the years, a better understanding of cancer pathogenesis has enabled the development of new therapeutic approaches, including targeted therapy, immunotherapy, stem cell transplantation, and hormone therapy [56,57].

These new approaches have provided alternative solutions to the side effects related to conventional treatments. Recently, an innovative approach has been represented by therapies based on the use of regulatory RNAs. This has been possible thanks to improved next-generation sequencing and recent advances in high-throughput sequencing technologies, bioinformatics analysis tools, and computational platforms, which have enabled researchers to study in greater depth the genomic and transcriptomic profiles of many human diseases, including cancer. These technologies have made it possible to identify and classify thousands of regulatory RNA molecules that have both oncogenic and tumor-suppressive roles in cancer. Overall, the deregulation of regulatory RNAs influences cancer development and progression through the modification of cellular processes such as cell proliferation, apoptosis, invasion, and metastasis [58].

The discovery of such a diverse number of regulatory ncRNA species has changed the way that researchers think about the physiology and development of diseases, which, for decades, has been focused on the study of protein-coding genes. Furthermore, due to the involvement of regulatory ncRNAs in every cellular process, their large numbers compared to proteins, their higher sensitivity and specificity compared to traditional tumor markers, and their easy detection in many body fluids, they can be considered a reservoir with incalculable potential, not only for the development of future therapeutic applications for cancer treatment and precision medicine but also in providing more effective tools for early cancer diagnosis or drug response prediction. For these reasons, cancer-focused clinical trials involving regulatory ncRNAs as novel biomarkers or therapies are increasing every year.

This review examines different classes of regulatory ncRNAs (miRNAs, siRNAs, piRNAs, lncRNAs, and cirRNAs), describing their biogenesis, functions, and clinical applications. The aim of this review is to provide not only the most complete overview possible of regulatory ncRNAs, in relation to the tumor cell biology, reporting both the positive aspects and the challenges to overcome for their use in clinical practice, but also to provide an indication of how research is evolving by describing clinical studies evaluating the use of regulatory ncRNAs in the oncology field.

## 2. Regulatory ncRNAs: General Overview

ncRNAs are a class of RNA molecules that are transcribed but not translated into proteins. Regulatory RNAs are a subclass of ncRNAs that are implicated in the regulation of gene expression. However, recent discoveries have rendered the classical definition of ncRNAs ambiguous, because many studies have shown that a certain number of ncRNAs harbor small ORFs that can encode micropeptides (less than 100 amino acids) [59], as in the case of “bifunctional RNAs”, which are so called because they can both function as ncRNAs and be translated into peptides [60,61,62]. These unconventional peptides play functional roles in normal and pathological processes, including cancer [63].

Regulatory RNAs represent a very large group of polynucleotides whose cataloging can be based on different and mostly arbitrary criteria, such as the structure of the ncRNA (linear, circular), their endogenous functions, and their lengths. The length is conventionally used to distinguish the two main subcategories of regulatory RNAs: small noncoding RNAs (sncRNAs), composed of RNA molecules with less than 200 nts, and lncRNAs, composed of transcripts longer than 200 nt [64,65,66] (Figure 1).

sncRNAs include, among others, miRNAs, siRNAs, and piRNAs; these represent the three pathways of RNAi. In general, RNAi is an evolutionarily conserved and sequence-specific mechanism that is triggered by dsRNAs. RNAi not only provides a defense mechanism against invading viruses and TEs but also plays a role in regulating gene expression at either the transcriptional or the post-transcriptional level. The conservation of this mechanism and the possibility and ease of designing dsRNAs have allowed RNAi to be exploited for gene therapy and clinical application in numerous diseases, including cancer.

Meanwhile, lncRNAs include lincRNAs, circRNAs, antisense RNAs, and pseudogenes; their functions are highly heterogeneous, as they may serve as sncRNA sponges, structural elements, decoys, antisense molecules, etc., on a case-by-case basis.

Functional analyses have revealed that regulatory RNAs are mainly involved in the control of gene expression, adopting different mechanisms, and they participate in virtually all cellular processes [67,68,69]. Considering their heterogeneous characteristics, it is plausible to suggest that, in perspective, the classification of ncRNAs might change because of the general improvement in investigation techniques and the better understanding of the mechanisms in which they participate. This will also help to reconcile the definition of “noncoding RNA” with the evidence that some ncRNAs can be translated into small peptides.

## 3. mtDNA and Cancer Implications

Human mtDNA is 16,659 bp long, has no histone support, and is composed of two strands, called the heavy (H) and light (L) strands, organized in a circular molecule. The mitochondrial genome is exclusively maternally inherited, and sperm mtDNA is not transmitted to the next generation [70,71].

mtDNA is present in the cell in multiple copies, with generally between 1 and 10 copies per mitochondrion [72]. However, the number of mitochondria per cell varies widely, as does the number of mtDNA copies, and this depends on the cell type and its energy needs [73,74,75,76,77].

Usually, the copies of mtDNA are all identical, a condition called homoplasmy. In contrast, heteroplasmy is the condition in which there is more than one mtDNA variant in a cell. For example, if mutations occur in one or more copies of mtDNA, a mixed population of mutant and wild-type genomes will coexist in the cell. Healthy copies of mtDNA can functionally complement the damaged ones up to a critical threshold depending on the type of mutation. Once this threshold is exceeded, the defect associated with the mtDNA mutation becomes manifest.

The mitochondrial genome harbors 37 intronless genes, including 2 rRNAs, 22 tRNAs, and 11 mRNAs (two of which are bicistronic) [78,79]. In addition, a portion of the mitochondrial 16s rRNA contains a small ORF that encodes a small peptide known as humanin, which has neuroprotective activity and is also implicated in carcinogenesis [80,81,82,83].

### 3.1. mtDNA Deregulation in Carcinogenesis

Many mitochondrial dysfunctions associated with carcinogenesis are attributable to mutations in nDNA. However, several studies have found mtDNA mutations in over 50% of the tumors analyzed [84,85,86,87]. Compared to nDNA, mtDNA is more prone to damage. In fact, recent studies showed that mtDNA has a 10- to 100-fold higher rate of de novo germline mutation than nDNA [88,89]. Accumulated damage to mtDNA causes mitochondrial dysfunction, often associated with the impaired functioning of respiratory chain complexes and intracellular signaling pathways, which drives the pathogenesis of a variety of human diseases, especially neurodegenerative disorders and cancer. The causes are multiple, including age, a higher mtDNA replication rate, and less effective mtDNA damage repair mechanisms. For example, as healthy cells age, they accumulate nDNA and mtDNA damage due to environmental exposure and cellular processes, yet the mechanisms that regulate this damage’s induction are still unclear [90,91].

#### 3.1.1. Replication and Repair Mechanisms Cause High mtDNA Mutation Rates

DNA polymerase gamma (PolG) is responsible for mtDNA replication and repair, and, until recently, it was considered the only polymerase present in the mitochondria. However, recent data suggest that several polymerases display activity in the mitochondria, such as polymerase theta (PolQ) [92]. Among the various DNA polymerases, PolG is the most reliable. Nonetheless, although mutations associated with its activity are rare, mtDNA replicates much more frequently than nDNA, which increases the likelihood of PolG-induced mutational events. In addition, PolG’s activity is influenced by over 300 point mutations that have been mapped in its coding gene and are associated with many inherited mitochondrial disorders [93].

PolQ is another polymerase that works in the mitochondrion and belongs to the family A DNA polymerases, like PolG. Fidelity measurements of PolQ revealed that it generates single-base-pair substitutions at a 10- to 100-fold higher rate than other characterized family members, making it one of the least reliable members of the family A DNA polymerases [94,95].

Damage to mtDNA, similarly to that to nDNA, can trigger the action of several mechanisms to ensure genome stability and guarantee the normal function of the mitochondria. In fact, in the mitochondrion, the mechanisms of excision repair, direct reversal, mismatch repair, and possibly ds break repair seem to be active. Instead, NER has not been confirmed as a system for mtDNA damage repair. However, these mechanisms seem less effective than those in the nucleus, and the key components of these pathways have not been characterized as well as those in the nuclear system [96].

For these reasons, the mtDNA sequence may contain SNPs, which, in some cases, have been correlated with carcinogenesis. For example, several mtDNA mutations are correlated with HCC progression, namely G3842A, which creates a premature stop codon in the mtND1 gene; A11708G, which results in amino acid substitutions in the mtND4 gene; and 12418insA, which result in frame shift mutations in the mtND5 gene [97]. In CRC, the mutations T4216C, T3394C, and C3497T cause an amino acid substitution, and the mutation 3565_3566insC causes a frame shift; these are associated with carcinogenesis and CRC progression [98,99,100,101,102,103]. mt-ND2 mutation G4776A enhanced the cell growth of HNC cells via the induction of HIF1α [104]. Yuan and coworkers analyzed the mutations of the mitND6 gene by sequencing the mtDNA of tumor tissue from 87 patients with primary LAC. The analysis identified eight missense mutations in the mtND6 gene, which resulted in amino acid changes, and three nonsense mutations in the same gene, which resulted in premature translation termination; these were significantly correlated with the pathological stage of the tumor, lymph node metastasis, and a shorter survival rate in LAC patients [105]. Beadnell et al. suggest that SNPs T3394C and C3497T in the MT-ND1 gene are correlated with distant metastasis [106].

Among mtDNA mutations, point mutations are the most frequent; however, mtDNA can be affected by different types of alterations that are linked to carcinogenesis. For example, the deletion from position 8470 to 13,447 in mtDNA, also known as the common deletion or mtDNA^4977^, is the most frequently observed deletion in human mtDNA. The common deletion is an important factor in the carcinogenesis of several tumors, including HCC [107,108], CRC [109,110], and brain tumors [111].

#### 3.1.2. Influence of mtDNA Copy Number in Tumors

An increased or decreased number of mtDNA copies, or CNV, is a condition often observed in tumors and correlated with cancer progression and severity. For example, Mennuni and Al-Awadhi suggest that high mtDNA levels accelerate the progression of LAC [112] and CC [113]. Alwehaidah et al. demonstrate that high mtDNA copy numbers play a significant role during the initiation of ThC [114]. In TNBC, cell proliferation and resistance to doxorubicin (a commonly used chemotherapy agent) are correlated with high CNV values [115]. Meanwhile, in BrC [116,117], brain tumors [72], bone cancer [118], cancers of the oral tract [119], and HCC [84,120], cancer progression and severity has been associated with a reduction in the mtDNA copy number. Finally, tumors have been described whose progression and severity can be related to either a decrease or an increase in CNV. For example, CRC tumors isolated from different patients and analyzed for CNV showed both conditions [121,122]. In general, the results obtained from the analysis of different tumors show very heterogeneous behavior in relation to CNV, and the regulatory mechanisms are partly unknown. Thus, understanding CNV’s contribution to carcinogenesis requires further efforts.

#### 3.1.3. Mitochondria and Numtogenesis Process

In some cases, a process called numtogenesis occurs, in which small fragments of mtDNA or the entire mitogenome are transferred into nDNA [123]. In this case, the mtDNA does not undergo mutations but instead becomes the cause of nDNA mutation. Indeed, the insertion into the nDNA leads to genetic instability through the destruction of regulatory sites or PC sequences. Numtogenesis can promote the onset of several pathologies, including cancer [22,124].

## 4. miRNAs

### 4.1. Biogenesis of miRNAs

**Figure 2:**
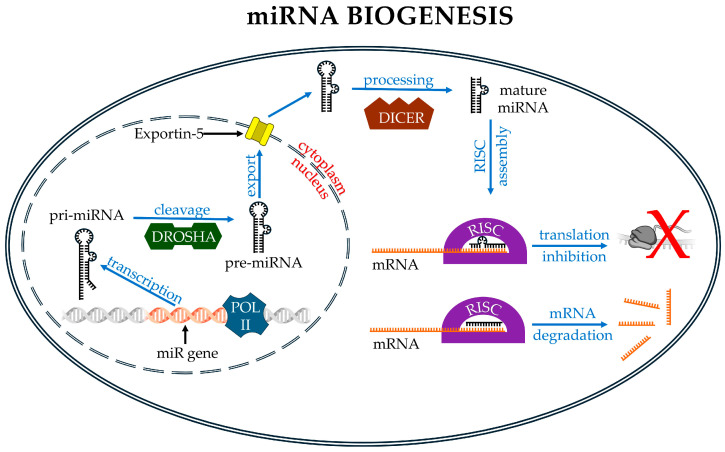
Main events in miRNA biology. In the nucleus, the gene containing the miR sequence is transcribed by RNA polymerase II, which produces a pri-miRNA. This molecule is then cleaved by Drosha to form a pre-miRNA, which is exported into the cytoplasm by Exportin-5. In the cytoplasm, the pre-miRNA is further processed by the DICER complex to produce a mature double-stranded miRNA. Upon loading into the RISC, one of the two RNA strand binds to its target mRNA, promoting either its translational repression (partial match, red X) or degradation (perfect match). Image partially built using freely available resources at NIH BioArt (https://bioart.niaid.nih.gov/).

The genomic sources from which miRNAs originate are multiple and differently organized. Indeed, miRNAs can be found not only within protein-coding genes—particularly in introns [125], at exon–intron junctions [126,127], or, more rarely, in exons [128,129,130]—but also in repetitive elements like TE [25,131,132] or in lncRNAs [129]. However, the origins of many miRNA molecules remain unknown [133]. Additionally, miRNAs can be organized as either single independent transcriptional units (annotated as miRNA host genes) or as multiple miRNAs embedded inside the same transcribed locus (Figure 2).

In the latter case, miRNAs form clusters whose transcription produces longer polycistronic primary miRNAs (pri-miRNAs), ranging in length from 1 to 10 kb. The pri-miRNAs contain the 5′ cap and the 3′ polyA tail and bear one or more hairpins, in which the mature miRNA sequence is located. The pri-miRNA is processed into a pre-miRNA in the nucleus by means of a complex known as the microprocessor. The microprocessor is a multiprotein complex in which Drosha and DiGeorge syndrome critical region 8 (DGCR8) constitute a minimal functional core. Drosha is a dsRNA-specific endoribonuclease III that binds at the stem–flank junction of the hairpin structure and mediates the cleavage of pri-miRNAs. DGCR8 forms a dimer that binds the terminal loop of the hairpin and interacts with Drosha, ensuring the accurate cleavage of pri-miRNAs [134,135,136]. The interaction between Drosha and DGCR8 induces a cleavage that generates a single pre-miRNA molecule with a length of approximately 55–70 nt. Pre-miRNAs are transported into the cytosol by the Exportin-5 (XPO5)/RanGTP complex. In the cytosol, pre-miRNAs are recognized by the Dicer/TRBP complex. Dicer, like Drosha, is a type III endoribonuclease. Dicer removes the loop structure from the pre-miRNA and forms mature miRNAs; these are ds segments whose lengths vary, according to different authors, in the range of 17–25 nts. The mature miRNA is loaded onto Argonaute (AGO) family proteins (AGO1–4), forming the pre-RNA-induced silencing complex (RISC). Within this complex, one of the two strands of the miRNA, the guide strand (antisense strand), is selected and retained, while the other, the passenger strand (sense strand), dissociates from the complex. The selection of the correct guide strand from the RNA duplex occurs through multiple mechanisms. For example, structural studies support a model that describes the thermodynamically asymmetric nature of duplex RNAs. Consequently, the strand with the most accessible 5′ end in the AGO binding pocket is the one that forms the thermodynamically most favorable bond and will function as the guide strand [137,138]. In addition, the identity of the 5′ nucleotide that specifically binds AGO can also influence the choice of the leader strand [139]. The RISC assembled with the guide strand becomes functional and is directed towards its targets by means of complementarity recognition between the guide strand and the target RNA. Additional non-canonical miRNA pathways exist and can be mainly distinguished as Drosha-independent or Dicer-independent pathways. For example, splicing and intron-debranching mechanisms can produce pre-miRNA-like structures that do not need to be processed by the microprocessor complex. These pre-miRNAs are then exported to the cytoplasm, where processing continues in the canonical pathway [140].

miRNA biogenesis using the non-canonical, Dicer-independent pathway is very rare. An example is the biogenesis of miR-451. Initially, pri-miR451 follows the canonical pathway in the nucleus: it is processed by Drosha and exported to the cytoplasm. However, the Drosha-mediated cleavage of pri-miR-451 produces a pre-miR-451 that is too short to be recognized by Dicer. Therefore, the cleavage of pre-miR-451 is mediated by AGO2. AGO2 has RNase-H-like endonuclease activity that can cleave specific miRNA precursors. Subsequently, the activity of a poly(A)-specific ribonuclease (PARN) is required to cleave the pre-miR-451 3′ end and produce mature miR-451. The functionality of miR-451 is dependent on its association with AGO2 [141,142].

### 4.2. Functional Role of miRNAs

The total number of miRNAs in the human genome is estimated to be several thousand. However, the most well-known miRNA databases, such as miRBasev.22.1 [143] and miRGeneDB v2.1 [144], report approximately 2000 miRNA molecules annotated for *Homo sapiens*. This discrepancy reveals the limited knowledge of miRNAs and their functions but, at the same time, suggests enormous potential that has not yet been explored. Interestingly, miRNAs can perform their activity either in the cell nucleus or in the cytoplasm.

In recent years, several studies have shown that miRNAs may perform functions within the nucleus, playing a role in transcriptional regulation. There, miRNAs can directly regulate miRNA biogenesis [145] or associate with target gene promoters [146] or enhancers [147,148].

Promoter-binding miRNAs and enhancers represent a subclass of miRNAs called NamiRNAs. NamiRNAs are transcribed by polymerase II from miRNA-coding genes with enhancer features. NamiRNAs help to assemble the complex containing polymerase II to allow the transcription of an activated enhancer. The resulting products are called eRNAs. NamiRNAs and eRNAs work together to activate gene expression [149,150].

In the cytoplasm, miRNAs are involved in gene regulation at the post-transcriptional level (Figure 2). Using different mechanisms, miRNAs can (I) promote the degradation of mRNAs using mechanisms such as deadenylation, decapping, and exonucleolytic decay [151]; (II) induce translational repression through the formation of the miRNA–RISC complex, which prevents the binding of the ribosome to the mRNA [152,153]; (III) bind other ncRNAs, thus indirectly controlling mRNA function. In particular, miRNAs may be part of a network consisting of various competing endogenous RNAs (ceRNAs), also called endogenous miRNA sponges. ceRNAs include various types of ncRNAs, such as lncRNAs or circRNAs. These ncRNAs likely prevent miRNAs from binding to miRNA-binding sites’ targets (miRNA recognition elements, or MREs) present in the mRNA [154,155,156].

miRNAs recognize and bind by sequence complementarity (Watson–Crick base pairing) to MREs present on different types of RNA molecules, including pseudogenes, lncRNAs, and circRNAs [157,158], and in the 3′ UTRs—or, more rarely, in the 5′ UTRs—of mRNAs [159,160,161,162]. The region of the miRNA complementary to the MRE is known as the seed sequence. The seed sequence represents a small part of the entire miRNA sequence and is usually included between nucleotides 2 and 7/8 from the 5′ end. The complementarity between the miRNA seed region and the MRE is responsible for the recognition of the correct miRNA target. Complementarity can be complete if all nucleotides of the seed sequence are involved in the interaction or partial if only some are involved. In partial interactions, bulges (structures formed when bases in one strand have no pairing partner in the opposite strand), wobble base pairs (a pairing between two nucleotides from two different strands that does not follow the Watson–Crick base pairing rule), or nucleotide mismatches (incorrectly paired nucleotides) may be present [163,164,165]. Moreover, a miRNA can bind its targets using additional mechanisms—for example, “pairing with the 3′ region” or using “centered sites”. Pairing with the 3′ region is a mechanism through which the region of the miRNA used for interaction with the mRNA is not limited to the seed sequence. In fact, this mechanism involves additional nucleotides located towards the 3′ end of the miRNA. This mechanism is used by miRNAs that use both full and partial pairing of the seed sequence [166,167]. Centered sites are non-canonical sites in miRNA targeting, consisting of contiguous base pairings of 11–12 nucleotides that occur starting from the third or fourth nucleotide and extending into the central region of the miRNA [168].

In general, the interaction between miRNAs and target RNAs is partial, since only a small part of the miRNA sequence is used. Furthermore, the involvement of few nucleotides increases the chance of a miRNA annealing on multiple RNA targets. This implies that a single miRNA may recognize many targets and, at the same time, allows a single target to be recognized by multiple miRNAs [58,169].

In addition to the well-known translational repression action, emerging evidence has revealed that some miRNAs can increase mRNAs’ stability and/or translation rate, resulting in target mRNAs’ upregulation [170,171,172]. The mechanism by which a miRNA induces gene upregulation is still partly unknown, but it seems favored under specific conditions. For example, some miRNAs, including let-7, activate translation during cell cycle arrest, but, in proliferating cells, they show the opposite effect [173,174]. Moreover, in quiescent cells, such as oocytes [175,176], or during amino acid starvation [177], it has been shown that miRNAs can upregulate gene expression.

The action of miRNAs may extend beyond the cell of origin thanks to their incorporation into exosomes. Exosomes are vesicles that are secreted by cells and are enclosed by a lipid membrane bilayer with a variable diameter of 30–150 nm. Exosomes transport many types of molecules, including proteins, lipids, DNA fragments, and different RNA species, including miRNAs. Thus, exosomes may connect two neighboring or distant cells by transporting messenger molecules. For example, it has been shown that exosomal miRNAs (exo-miRNAs) participate in various processes of tumorigenesis, including (but not limited to) tumor invasion and metastasis [178,179,180], cell proliferation [181], angiogenesis [182], and EMT [183,184]. Additionally, exosomes derived from tumor cells act as messengers and control tumor cells’ behavior within the tumor microenvironment [185]. Exo-miRNAs can influence the cancer treatment response as well [186,187,188]. Exo-miRNAs are primarily studied for their potential use as non-invasive biomarkers but represent an exciting frontier in cancer therapeutics research. However, to date, their translation into clinical practice poses significant challenges and requires further investigation.

Finally, a particularly interesting aspect concerns exogenous miRNAs or xeno-mirs. Cells, in addition to endogenous miRNAs and those received through exosomes, can contain miRNAs that are derived from other organisms and are mainly taken in through the diet. Xeno-mirs can influence cellular functions and play a role in maintaining the health of the organism [189,190,191]. In fact, some xeno-mirs act on the functions of the gut microbiota, and this, in turn, can play a role in the development of pathologies including coronary artery disease, neural degenerative diseases, and cancer [190,192]. The role of miRNAs in cancer was identified for the first time in 2002 by Callin and collaborators, who demonstrated that miR-15 and mi-R16 map at chromosome 13q14, a region frequently deleted in CLL. This deletion causes the absence or downregulation of both miRs in the majority of CLL cases [193].

In every type of cancer analyzed so far, the anomalous expression of several miRNAs has been observed, and, depending on their targets, miRNAs can act as either oncogenes or tumor suppressors [194,195].

### 4.3. Therapeutic Applications of miRNAs

Among ncRNAs, miRNAs are the most investigated in cancer, and their broad role in tumorigenesis makes them excellent candidates for the development of new and personalized therapeutic strategies. Nonetheless, several drawbacks limit their use in clinical practice. The lack of an effective delivery system capable of protecting RNA molecules from degradation by nucleases is one of the main issues. In addition, a molecular transport system that guarantees their release specifically in tumor cells, without inducing adverse effects such as an excessive immune response, is still a problem in the design and delivery of these molecules.

At present, miRNA-based therapeutics involve either miRNA inhibition or miRNA replacement [196]. In the first approach, chemically modified ASOs, such as LNAs or Antagomirs, are used, while, in the second approach, miRNA mimics are used. An important consideration is that miRNAs act as inhibitors of gene expression; therefore, strategies that aim at their inhibition have the effect of activating the expression of the target gene of the miRNA [197].

**Figure 3:**
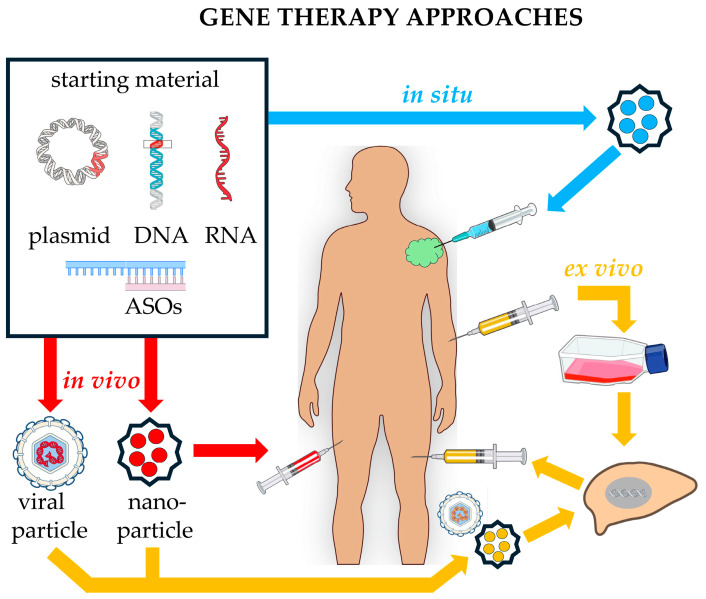
Comparing different approaches in gene therapy. In the in vivo approach (red arrows), the starting material is incorporated into a vector (a viral or nanoparticle) and then injected into the patient. In the in situ approach (blue arrows), using appropriate vectors, the starting material is directly injected into the site of interest (e.g., a tumoral mass), where it exerts its effects. In the ex vivo procedure (yellow arrows), cells are explanted from the patient and cultured in vitro. Upon growth and selection, some cells are transformed using appropriate DNA vectors, such as a virus, to insert the sequence of interest into recipient cells, which are then transplanted back to the same donor. Image built using freely available resources at NIH BioArt (https://bioart.niaid.nih.gov/).

ASOs represent a large and heterogeneous group of ss DNA or RNA molecules, of approximately 15–21 chemically modified nucleotides [198], that bind to complementary miRNA sequences, modulating their functions (Figure 3).

Chemical modifications endow ASOs with characteristics such as stability and cellular availability, target affinity, and cellular uptake, making them superior to sequences consisting of only canonical oligonucleotides. In fact, canonical oligonucleotides are easily degraded by both serum exonucleases and intracellular endonucleases, a condition that strongly impairs their therapeutic efficacy. Depending on their chemical modifications, ASOs have evolved through three generations. The first generation consists of ASOs with modifications of the phosphodiester bond. In these, one of the free oxygens of the phosphate group is replaced by a specific chemical group (sulfur, methyl, or amine group), generating the PSs, methylphosphonates and phosphoramidates. PSs represent the most widely used group of first-generation ASOs since this chemical modification confers an improvement in the stability of the structure, with consequently increased resistance to nuclease degradation and the elongation of its half-life. Members of the first generation can activate an RNAse H response; this is a ubiquitous enzyme that cleaves the RNA strand in a DNA–RNA duplex. In the case of ASO treatment, the activation of RNAse H allows the degradation of the target RNA within the ASO/RNA complex. Despite these positive aspects, first-generation ASOs are toxic and poorly specific [199]; consequently, researchers have explored other types of chemical modifications, which has led to the emergence of second-generation ASOs.

The second generation of ASOs consists of alkyl modifications at the 2′-position of the ribose, which leads to the formation of 2′-O-methyl and 2′-O-methoxyethyl nucleotides [200,201]. These modifications, on the one hand, improve their specificity and decrease their toxicity, but, on the other hand, they inhibit the ability to activate RNAse H. Therefore, second-generation ASOs are useful in cases where transient inhibition but not RNA degradation is required. The third generation is, in contrast, very heterogeneous in terms of the chemical modifications tested. This allow us to improve, depending on the chemical modification used, different characteristics, such as the binding affinity, nuclease resistance, pharmacokinetics, and thermal stability of ASOs. Example of these molecules are reported below.

#### 4.3.1. LNAs in miRNA Inhibition-Based Therapy

One of the most widely used molecules belonging to third-generation ASOs is the LNA. LNAs are DNA or RNA sequences formed from a ribose sugar moiety modification in which the 2′-oxygen is connected to the 4′-carbon through a methylene bridge [202,203]. The structure of an LNA possesses characteristics that are useful for its use as a therapeutic agent, such as stability under nuclease-mediated degradation, excellent sequence specificity, good solubility in the aqueous phase, low toxicity, and high stability both in vivo and in vitro [204]. In relation to the structure of the molecule, synthetic LNAs are divided into two main groups: mixmers and gapmers. Mixmers are oligonucleotide sequences formed by LNA and DNA nucleotides that are randomly placed next to each other. Gapmers are also oligonucleotide sequences but, in this case, the DNA nucleotides are located in the center of the sequence, while the LNA nucleotides are found on either side [202]. Mixmers and gapmers can bind both DNA and RNA, and this makes their use particularly versatile. In general, LNAs work using different mechanisms of action: (I) they can induce the destruction of the target (activating RNase H or the RISC), (II) they can cause splicing alterations, or (III) they can induce a steric blockage in the target RNA.

The activation of RNase H is obtained using gapmers. Indeed, the DNA sequence contained within the LNA gapmer forms a DNA/RNA hybrid. This hybrid activates RNase H, with the consequent destruction of the target RNA [205,206]. Helmen and collaborators described the use of molecules consisting of a combination of mixmers and siRNAs, called siLNAs. siLNAs are not only compatible with the intracellular machinery of siRNAs but also mediate the activation of the RISC and the destruction of their target RNAs. Notably, siLNAs show a longer serum half-life than unmodified siRNAs [207].

LNAs can be used to induce alterations in the splicing process. In fact, their binding to the splice site at an intron/exon boundary can block the splicing of a specific intron and thus direct the splicing process towards the production of a specific product [208]. This use is particularly interesting because the relative abundance of alternatively spliced mRNA variants in tumor cells is different from that in healthy cells, suggesting an important contribution of mRNA alternative splicing in carcinogenesis [209].

A further mechanism of action promoted by LNAs is steric blockage, i.e., the reversible attack of the LNA molecule to the complementary miRNA. Obad and collaborators describe a method in which short LNA sequences (8-mer LNA oligonucleotides) can simultaneously inhibit multiple miRNAs that share the same seed sequence, with the concomitant upregulation of direct targets [210].

#### 4.3.2. Antagomirs in miRNA Inhibition-Based Therapy

Antagomirs are a group of ASO-derived anti-miRNAs, also known as anti-miRNA ASOs or blockmirs, that function by binding complementary miRNAs, thus inhibiting their action on target genes. Antagomirs are characterized by a sequence consisting of ssRNA analogs conjugated to cholesterol. In vivo laboratory tests on murine models showed that the use of cholesterol in the antagomir improves its cellular delivery compared to other ASOs. In addition, antagomirs possess some good pharmacological characteristics, such as specificity, efficiency, and prolonged effects [211].

An example of their therapeutic use is described in the work of Wang J. and collaborators [212], who used miR-BART1-5p-antagomirs to inhibit a specific miRNA produced by EBV. EBV produces different miRNAs that can be secreted via exosomes from infected cells and affect the tumor microenvironment. Among these miRNAs, miR-BART has been associated with growth and invasion in several types of tumors, such as Hodgkin lymphoma and GC. In nasopharyngeal carcinoma EBV-miR-BARTs are associated with the mechanisms of VM, i.e., the process that leads to the formation of microvascular channels composed of tumor cells, and angiogenesis. The authors generated a therapeutic targeting exosome system with miR-BART1-5p-antagomirs and observed an inhibitory effect on both VM and angiogenesis. This effect is probably due to the increase in the expression levels of proteins such as Ras, c-Raf, MAPK, VEGF, PI3K, Akt, mTOR, and HIF1-α, which are important effectors of the signaling pathways that regulate angiogenesis and VM [212].

#### 4.3.3. miRNA Replacement Therapy

miRNA replacement therapy is based on the use of synthetic miRNAs (also known as miRNA mimics), whose function is to re-establish the normal levels of expression and function of a specific endogenous miRNA that is underexpressed in tumor cells [213]. There are several types of mimics, which differ in their structure and the type of nucleotide chemical modifications used in the synthesis of the miRNA molecule [214,215]. However, both the initial steps required for the development of miRNA mimics and the chemical modifications of the nucleotides used are not well defined and are often covered by intellectual property rights [214].

The mechanism of action used by miRNA mimics involves their loading onto RISC and silencing their target mRNAs through the normal miRNA signaling pathway. The miRNA mimics used to activate RISC can be either ss or ds. In the case of ss (miRNA precursors), the mimic contains a sequence identical to the guide strand of the mature miRNA. The miRNA precursors can be both pri-miRNAs and pre-miRNAs. The pri-miRNA is transfected into cells and enters the nucleus, where it undergoes the first processing step. It is then translocated into the cytoplasm, where it is cleaved by Dicer and transformed into a mature miRNA. Instead, the pre-miRNA, after entering the cell, is directly cleaved by Dicer [214,216]. If ds, the miRNA mimic contains both the guide strand and the passenger strand. A comparison of ss miRNAs with ds miRNAs shows that ds miRNAs are 100 to 1000 times more effective than ss miRNAs [196,217]. The difference in efficacy observed between ss and ds miRNAs is due to the ds structure, which can facilitate the correct loading of the RNA molecule into the RISC, thus enhancing the gene silencing effect. Therefore, the design of mimetic miRNAs with a duplex structure, such as agomirs (see below), has become a major direction in therapeutic development.

Agomir miRNAs are short ds artificial RNAs in which the antisense strand has the same chemical modifications described for antagomirs. The applied chemical modifications allow not only higher affinity for the cell membrane, consequently increasing the transfection efficiency, but also greater intracellular enrichment compared to other types of miRNA mimetics, due to better resistance to degradation and greater stability in cells [218].

Overall, the use of miRNA mimics as potential therapeutic agents in cancer is still in the early stages of clinical development, but their potential as drugs is clear.

### 4.4. miRNA-Based Therapies in CTs

CTs evaluating ASOs and their derivatives as a possible therapeutic strategy in the oncology field are increasing. Their use is mainly based on their ability to selectively target mRNAs and consequently silence cancer-associated proteins [219,220,221]. In this way, pathogenic processes associated with the protein function are interrupted at the molecular level. However, their use towards ncRNAs, such as miRNAs, is still very limited. In fact, a search on the ClinicalTrials.gov website, which is a database of clinical research studies conducted around the world, including their results, for therapies that use miRNAs as a therapeutic target produces only eight results to date (Table 2). These studies mainly use LNA and miRNA mimetics as therapeutic tools.

NCT04811898 was a trial completed in 2021, in which a 13-mer LNA inhibitor of miR-221 (LNA-i-miR-221) with a full PS-modified backbone was analyzed for its safety and tolerability in patients affected by refractory multiple myeloma and advanced solid tumors. This LNA downregulates miR-221 and upregulates its targets, i.e., CDKN1B/p27 and PTEN. The study demonstrated that LNA-i-miR-221 has an excellent safety profile and anti-tumor activity, thus representing the first clinical evidence of the use of an LNA for the treatment of tumors [222].

The drug MRG-106 (cobomarsen) is an LNA inhibitor of miR-155 that stops cell proliferation and induces cell apoptosis in MF-CTCL cell lines and HTLV-1^+^ CTCL cells. MF-CTCL is a type of non-Hodgkin lymphoma localized in the skin; it is the most common form of cutaneous T-cell lymphoma. HTLV-1 is a virus that infects T cells and can cause leukemia and lymphoma. HTLV-1^+^ CTCL cells are CTCL cells infected by the HTLV-1 virus. Cobomarsen has been used in three clinical studies: NCT02580552, NCT03713320, and NCT03837457. NCT02580552 was a phase 1 CT completed in 2020. In this trial, cobomarsen was tested in MF-CTCL, CLL, DLBCL, and ATLL [223,228]. Given the successful results of the phase 1 CT in terms of clinical safety, efficacy, and pharmacokinetics, an additional CT (NCT03837457—phase 2) using the same drug was carried out. The primary aim of this second CT was to investigate the efficacy and safety of cobomarsen for the treatment of MF-CTCL in subjects who had confirmed disease progression following treatment with Vorinostat in the SOLAR clinical study (MRG106-11-201). The trial was terminated in 2020 with the following justification: “study no longer needed because eligible subjects may receive treatment with cobomarsen in a crossover arm of the SOLAR CT (NCT03713320)” [229]. The phase 2 CT NCT03713320 was the third trial involving the use of cobomarsen. The primary objective of the trial was to study the efficacy and safety of this molecule for the treatment of MF-CTCL. The study aimed to compare the effects of cobomarsen and Vorinostat, a previously approved drug used for the treatment of CTCL. Unfortunately, the trial was terminated early for business reasons and not due to concerns regarding cobomarsen’s safety or efficacy [230].

CT NCT01829971 was the first-in-human, phase 1 study of a miRNA-based cancer therapy. The purpose of this trial was to evaluate the safety of the drug MRX34, which is a synthetic ds miR-34a mimic encapsulated in a liposomal nanoparticle. Patients participating in the trial received at least one dose of MRX34 intravenously. Patients had solid tumors, including HCC, Me non-cutaneous excluding uveal, SCLC, TNBC, Sa, BlC, RC, and OC. The trial was terminated early in 2017 because five immune-related serious adverse events occurred among the 85 patients studied, resulting in four patient deaths [224,231,232]. The MRX34 drug was also evaluated in a phase 1B CT, NCT02862145. This trial involved the use of MRX34 combined with dexamethasone in Me cancer patients. The trial aimed to investigate the biomarkers, pharmacodynamics, and pharmacokinetics of MRX34. The participants were Me patients with easily accessible lesions who were monitored through serial biopsies and serial blood sample collection. Consequent to the adverse events observed during the first trial, the NCT02862145 trial was withdrawn in 2017 before participants were enrolled. However, despite the side effects, it was observed that treatment with MRX34 decreased the expression of miR-34 target genes, oncogenes, and immune escape genes in cancer patients. Therefore, miR-34a is still a promising target for miRNA-based cancer therapy.

The phase 1 CT NCT02369198 was the first human trial of the drug Targomir. The trial aimed to evaluate the safety and activity of Targomir in MPM and advanced NSCLC. Targomir is a new technology in the context of miRNA mimic-based therapies [225,233]. It consists of three parts: (a) a miRNA mimic based on miR-16, as several different forms of cancer have been linked to the miR-16 family’s role as tumor suppressors [234]; (b) a drug delivery system called EnGeneIC Dream Vector (EDV), where EDVs are non-living bacterial mini-cells (nanoparticles) that enable the efficient packaging of drugs, proteins, or nucleic acids inside them; (c) as a targeting moiety, an anti-epidermal growth factor receptor (EGFR) antibody that directs the EDVs to cancer cells expressing EGFR [225,233]. Indeed, it is known that EGFR is consistently deregulated in both LC and mesothelioma; it can therefore be used to target Targomir specifically to tumor cells [235,236]. NCT02369198 was completed in 2017 and involved 27 patients, of whom 26 received at least one dose of Targomir (one patient died before starting the treatment). During the trial, 21 deaths occurred, of which 20 were related to tumor progression and one was due to bowel perforation (caused by a second primary tumor). The experimentation allowed the researchers to establish not only the maximum tolerated dose but also the early signs of anti-tumor activity in patients with MPM [233]. Overall, this study offered new hope for mesothelioma patients, of whom less than 10% currently survive for more than 5 years [237]. Furthermore, the positive results obtained support a future phase 2 CT to evaluate the efficacy of Targomir therapy alone or in combination with conventional chemotherapy.

The drug INT-1B3 is an LNP-formulated miR-193a-3p mimic that was evaluated in the phase 1/1B CT NCT04675996. This trial was a first-in-human clinical study aiming to evaluate the safety, pharmacokinetics, pharmacodynamics, and preliminary efficacy of INT-1B3 in the treatment of patients with advanced solid tumors. In previous preclinical work, the function of synthetic miR-193a-3p mimic 1B3 was tested in cell lines derived from several cancers, such as TNBC, NSCLC, Me, CRC, and HCC. Treatment with 1B3 resulted in the upregulation of the tumor-suppressive PTEN pathway and the downregulation of many oncogenic pathways in cancer-derived cells. In addition, despite the different genetic backgrounds of these cancer cell lines, 1B3 showed consistent effects in suppressing cell proliferation, cell cycle progression, and cell migration and inducing apoptosis, cell senescence, and DNA damage. These results suggest the potential of IB3 in a broad range of cancers. The NCT04675996 trial started in 2020, and the last study records uploaded to the ClinicalTrials.gov website were provided in February 2024; the trial is currently described as “terminated due to insufficient funding” [226,238].

### 4.5. Recent CTs Evaluating miRNAs as Biomarkers

To date, the most numerous clinical trials based on miRNAs are those evaluating their possible use as tumor markers, as reviewed by Kim and Croce [239]. Therefore, this review aims to provide up-to-date information on the most recent clinical trials, registered on the ClinicalTrials.gov website, that have used miRNAs as potential diagnostic or prognostic biomarkers in various types of cancer (Table 3).

NCT06738225 is a CT that will start in 2025 and evaluate miR-15b and miR-21 as diagnostic biomarkers of CRC [240] by comparing their expression levels in CRC patients and healthy individuals.

NCT06610851 and NCT06203496 are CTs that seek to improve the knowledge of GB recurrence. NCT06610851 is a study initiated in 2024 that aims to identify miRNAs that can be used to monitor patients undergoing surgery for grade 2 and 3 GB, thus allowing the early diagnosis of recurrence. GB represents tumors of the central nervous system and is divided into four histological grades of malignancy. The treatment of GB is based on tumor removal and radiotherapy/chemotherapy treatments, depending on the grade of the glioma and the quality of the excision. In the case of recurrence, to be detected as early as possible, the patient can receive second-line chemotherapy. However, for grade 2 and 3 GB, monitoring is imperfect because it is not possible to detect tumor recurrence at an early stage. For these reasons, the use of early biomarkers, such as miRNAs, to monitor patients with grade 2 and 3 GB allows the timely diagnosis of recurrence. NCT06203496 focuses on changes over time in the plasma levels of pro-oncogenic miRNAs, after the surgical removal of a grade 4 GB, to assess whether they can be used to identify false-positive recurrences on magnetic resonance imaging.

In CT NCT06730035, EV circulating in plasma and the miRNAs that they contain are analyzed. The aim is to observe whether changes in the content of EV during neoadjuvant radiotherapy for locally advanced rectal tumors could provide early indications of the tumor’s response to treatment. Neoadjuvant radiotherapy is generally performed before surgery to reduce the size of the tumor to be removed. Therefore, obtaining early information on the response to neoadjuvant radiotherapy could contribute to the choice of a personalized therapeutic strategy.

In the observational study NCT06702891, the researchers are looking for specific biomarkers and potential therapeutic targets that can be used to develop new diagnostic tools and treatment strategies for GcC. GcC is a type of stomach cancer that begins in the mucus-producing cells in the lining of the gastric cardia (the part of the stomach closest to the esophagus). In this study, clinical information is collected from multiple biological samples, such as serum and tissue, which will be analyzed using an integrative analysis consisting of exosome-mediated single-cell transcriptomics and proteomics.

Additional clinical trials are currently running, for which only a very limited number of data is available. We recall here the following. NCT06224166 is a multicenter study in HNC patients, where miRNA detection from blood and saliva samples is being used as a non-invasive strategy to detect HNC recurrence. NCT06001099 is a prospective study to validate miRNAs, extracted from blood samples, for the early diagnosis of gynecological tumors. The aim of the NCT05901376 trial is to verify whether miR-20a, miR-21, miR-106b, miR-199a, and miR-22, extracted from blood samples, are upregulated in GC patients compared to healthy volunteers, to be effectively used as diagnostic biomarkers. The NCT06240195 trial is a prospective, multicenter study aimed at identifying predictive biomarkers of the efficacy/tolerability of Sacituzumab–Govitecan in the treatment of patients with metastatic TNBC. Sacituzumab–Govitecan is an antibody–drug conjugate composed of an antibody targeting human trophoblast cell surface antigen 2 (Trop-2), expressed in most breast tumors, coupled to SN-38 (a topoisomerase I inhibitor) through a hydrolysable linker [241].

The current recruitment status of the 2023 trial NCT05697224 is “not yet recruiting”. NCT05697224 aims to evaluate a *Schistosoma haematobium* (*Sch*)-specific miRNA, Sha-miR-71a, as a potential marker for the early diagnosis and prognosis of bilharzial BlC. An analysis of urine from patients with bilharzial BlC shows higher levels of Sha-miR-71a compared to both those observed in BlC not associated with bilharziasis (schistosomiasis) and in benign bladder cystitis associated with schistosomiasis [242]. This observation suggests that Sha-miR-71a can be used in the identification of BlC associated with infection. *Schistosoma haematobium* is a trematode worm that causes parasitic disease. In particular, the *Sch* eggs trapped in tissues release antigens that induce an immune response called schistosomiasis, or bilharzias/bilharziasis, in honor of the German surgeon Theodore Bilharz, who first identified the etiological agent *Sch* in 1851. The persistent immune response leads to the formation of granulomas, a compact assembly of inflammatory and resident cells, e.g., T cells, macrophages, and eosinophils, which form a well-defined structure surrounding the parasite eggs. A granulomatous reaction can result in organ damage [243]. In addition, urinary bladder infection due to *Sch* is correlated with the induction of BlC; in fact, the International Agency for Research on Cancer considers *Sch* a biologically carcinogenic agent [244].

The CT NCT05746858 aims to identify biomarkers that will predict the outcomes of standard and targeted therapies in patients with relapsed/refractory DLBCL.

Finally, the last CT, started in 2023, is NCT06320184. The goal of this CT is to refine LC risk assessment using blood biomarkers, including circulating miRNAs, in combination with artificial intelligence (AI)-integrated low-dose computed tomography to further implement LDCT screening strategies.

## 5. siRNAs

### 5.1. Biogenesis of siRNAs

siRNAs’ biogenesis can start from either exogenous or endogenous sources. Exogenous sources are predominantly foreign nucleic acids such as cytoplasmic dsRNAs derived from viral genome replication upon infection or from RNA secondary structures within viral genomes. Endogenous sources are specific genomic regions, such as repetitive DNA or TE, whose transcription gives rise to siRNA precursors. Mature siRNAs that originate from an endogenous source are called endogenous siRNAs or endo-siRNAs. Both endogenous and exogenous sources initially generate a long dsRNA precursor that will be processed to produce the mature siRNA.

Although the genomic origin of endo-siRNAs has been described in many model organisms, including *Caenorhabditis elegans* [245,246], *Drosophila melanogaster* [247,248,249,250], and *Mus musculus* [251,252,253,254,255,256], in *Homo sapiens*, the genomic origin of siRNAs remains elusive. However, the results presented in the work of Chen and collaborators suggest that the expression of the LINE-1 TE is regulated by endo-siRNAs. Indeed, in human BrC cells, both the significant depletion of endo-siRNAs and the increased activity of LINE-1 are observed compared to normal breast cells. The overexpression of endo-siRNAs in BrC is correlated with the silencing of endogenous LINE-1 expression [257]. Additionally, Jing and coworkers, building a small RNA deep sequencing data set, identified endo-siRNAs derived from tandem Alu SINE TE within the intron of the gon-4-like (GON4L) gene [258].

The long dsRNA precursor of siRNA is processed similarly to miR (Figure 2); in the cytoplasm, Dicer cleaves the precursor into a smaller dsRNA molecule known as a siRNA. siRNAs are approximately 21–23 nt long, with two-nucleotide overhangs at the 3′ end. The siRNA is then loaded into the RISC, where the endonuclease AGO2 cleaves the passenger strand of the siRNA, while the guide strand remains associated with the RISC. The siRNA guide strand directs the active RISC to its target mRNA for cleavage by AGO2 [259].

### 5.2. Functional Role of siRNAs

The functional mechanism of siRNAs shares some elements with the miRNA pathway, such as the involvement of the AGO2 protein and the formation of the siRNA–RISC complex, which induce the consequent endonucleolytic cleavage of siRNA targets (Figure 2). The sharing of some elements between the siRNA and miRNA pathways implies that the use of siRNAs as drugs requires the optimization of their concentrations to avoid the saturation of the RISC and the alteration of the endogenous mechanisms controlled by miRNAs [260,261,262]. The main functional difference between miRNAs and siRNAs is that the latter exploit fully complementary base pairing with their targets, i.e., mRNAs, lncRNAs, and circRNAs [263,264]. This feature allows siRNAs to recognize only a specific target.

In addition to the mentioned post-transcriptional silencing occurring in the cytoplasm, siRNAs and some components of their pathway, such as AGO2 and Dicer, have also been found in the nucleus. Nuclear siRNAs bind to promoter regions and mediate chromatin remodeling and histone modifications, resulting in transcriptional silencing [265]. Synthetic siRNAs are commercially available and have been widely adopted in RNAi technology. The use of RNAi with siRNAs allows the silencing of specific targets, thus representing another strategy to modulate overexpressed RNA molecules in cancer [266].

### 5.3. Therapeutic Applications of siRNAs

In the context of clinical applications, siRNAs and miRNAs share not only the same limitations, such as poor stability in vivo, delivery challenges, immune responses, and off-target effects, but also strengths, as their nature allows the development of similar strategies aimed at enhancing their efficacy and specificity, thus reducing off-target effects. siRNAs are generally introduced into cells via transfection, but their effects are transient because they are rapidly degraded. However, it has been observed that some chemical modifications, such as the substitution of the ribose 2′-OH group with other chemical groups (including 2′-O-methyl (2′-O-Me), 2′-fluoro (2′-F)) or the use of siRNAs composed of LNAs, increase their stability [207,267,268]. Furthermore, siRNAs can cause immune responses in both sequence-independent and -dependent manners. In the first case, through the activation of protein kinase R, many genes belonging to the interferon pathway are stimulated, which is part of the defense mechanism against viral infection, resulting in non-specific mRNA degradation and apoptosis. In the second case, specific immunostimulatory sequences induce the activation of the immune response by activating the transmembrane receptors TLR 7 and TLR 8 present in the endosomes of immune cells. Possible solutions to these problems include, for example, the use of delivery agents that exclude the endosomal release of siRNAs (electroporation), the replacement of immunostimulatory sequences with other sequences that do not induce such a response, and the use of chemically modified immunostimulatory sequences that do not allow their recognition by TLR receptors [269]. However, the modifications made to siRNAs can have toxic effects or render the molecule less efficient; therefore, the use of these substances in the clinical setting must be carefully evaluated to avoid adverse effects in patients [270].

**Figure 4:**
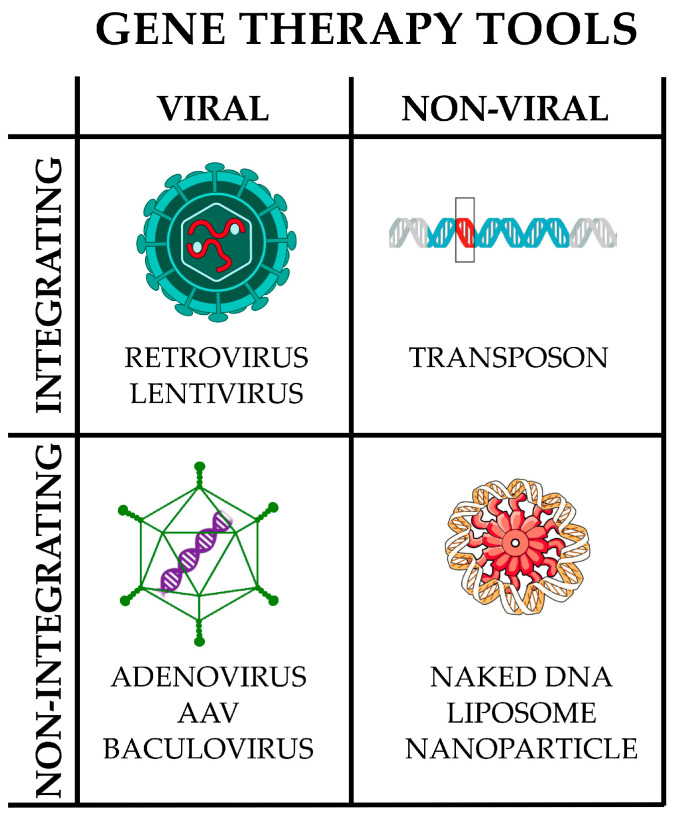
Main gene therapy tools. They can be broadly divided into viral and non-viral; in turn, each may or may not integrate into the host genome. Only representative examples are reported; the list is not intended to be comprehensive. Image partially built using freely available resources at NIH BioArt (https://bioart.niaid.nih.gov/).

A strategy to improve siRNAs’ efficiency is the use of short hairpin RNAs (shRNAs), which are expressed in the nucleus through delivery by viral vectors (Figure 4).

Within the nucleus, shRNAs are transcribed by RNA polymerase III, bypass Drosha processing, and are exported to the cytoplasm via XPO5 as pre-miRNA-like molecules. In the cytoplasm, they enter the physiological siRNA processing pathway, which culminates with loading into the RISC [271]. The viral presentation method, which includes the use of lentiviruses, adenoviruses, and adeno-associated viruses [272,273,274], guarantees not only very high efficiency in transferring the shRNA vectors into the nucleus but also high shRNA expression. The use of lentiviruses, which can integrate into the host genome, allows for shRNA-based therapies that are more persistent than siRNA-mediated ones, which require repeated administration because they are dependent on the rate of cell division. However, integration into the host genome increases the risk of insertional mutagenesis [275,276]. Furthermore, the use of viruses, particularly adenoviruses, is associated with high immunogenicity [277]. Recently, Alsing and collaborators developed a novel system, used for retinal gene therapy, that uses lentiviral vectors to present an expression cassette transcribed by polymerase III consisting of an RNAi construct (VEGFA-RNAi) in an Ago2-dependent shRNA (agshRNA) vector. The agshRNA vector is designed to be processed by Ago2 and produces a single guide strand, while classical shRNAs are processed by Dicer into a guide strand and a passenger strand. Since agshRNAs produce no passenger strand activity, a decrease in undesirable cellular responses is observed. Furthermore, agshRNAs show increased specificity and safety compared to shRNAs [278,279].

### 5.4. siRNA-Based Clinical Studies

The first CT involving the use of siRNAs dates back to 2004. In that trial, siRNA-027 was used for the treatment of age-related macular degeneration [280]. Meanwhile, the first clinical study using siRNAs for the treatment of cancer began in 2008 (NCT00689065) [281,282,283], with a phase 1 CT for the treatment of several solid tumors. The trial evaluated not only the safety, toxicity, and MTD but also the tumor response to the drug CALAA-01, a nanocomplex consisting of four main components: a siRNA duplex, a polymer, a stabilizing agent, and a targeting agent. The siRNA duplex used was not chemically modified and was designed to reduce the expression of the Ribonucleotide Reductase M2 subunit (RRM2), which participates in nucleotide metabolism and catalyzes the conversion of nucleotides to deoxynucleotides, maintaining dNTP pools for DNA biosynthesis, repair, and replication [284,285]. The other three components (the polymer, the stabilizing agent, and the targeting agent) form a nanoparticle of approximately 100 nanometers in diameter, inside which the siRNA is transported, thus ensuring its protection against nuclease degradation. The polymer is the basic constituent of the particle and it is a cyclodextrin-based polymer. The stabilizing agent is a hydrophilic polymer, PEG, used to promote nanoparticle stability in biological fluids. Finally, the targeting agent is constituted by human transferrin protein (Tf), which is exposed on the surface of the nanoparticle. Tf is recognized by Tf receptors (TfR) on the surfaces of cancer cells that overexpress the receptor. When CALAA-01 reaches the target cell, Tf binds to the TfRs on the cell surface, inducing drug endocytosis. Inside the cell, the nanoparticle releases a siRNA, which can exert interfering effects on RRM2. Data obtained from the trial revealed slight liver toxicity due to the chemical nature of the nanoparticle constituents but not to the siRNA used. Toxicity was alleviated by using a predosing hydration protocol (500 mL of 5% (wt/vol) dextrose in water before CALAA-01 infusions) and by advising patients to drink 2–3 L/day of fluids during treatment. However, this study was terminated early because 7 of 24 patients enrolled in the trial (29%) experienced disease progression characterized by an increase in tumor size [281].

Nowadays, there are many clinical studies using siRNAs as a therapeutic tool in oncology, but, in this case, similarly to what was described for miRNAs, the preferred targets are the overexpressed mRNAs of cancer-associated proteins [219,221].

Meanwhile, the use of siRNAs directed against specific ncRNAs is still in the preclinical stage. In particular, siRNAs are used to reduce the expression of overexpressed ncRNAs in tumors and evaluate the effects of silencing in relation to both the response to pharmacological treatment and improvements in tumor hallmarks (proliferation, migration, invasion, apoptosis, epithelial–mesenchymal transition).

However, there are some particularly interesting preclinical studies, such as the works of Liu [286] and Connerty [287], which used siRNAs to silence lncRNAs or circRNAs; these may soon be evaluated in CTs. Liu et al. tested a trivalent N-acetylgalactosamine (GalNAc)-conjugated siRNA construct, named GalNAc-silncRNA16 or Nano-silncRNA16, to perform the silencing of lncRNA16. An analysis of the serum lncRNA16 levels in NSCLC patients suggested that patients with elevated lncRNA16 values showed a poor response to chemotherapy, implying that lncRNA16 is a possible therapeutic target. Preclinical studies in mouse models of NSCLC suggest that silencing lncRNA16 with GalNAc-silncRNA16 restores chemosensitivity and results in tumor growth inhibition. Furthermore, GalNAc-silncRNA16 is specific and without detectable toxicity [286]. In the study conducted by Connerty et al., an LNP formulation, D-Lin-MC3-DMA, was used to deliver a siRNA for the treatment of t(8;21) pediatric ALL. This construct silences LINC01257, which is an oncogenic lncRNA that is overexpressed in AML cells, resulting in rigorous tumor growth and differentiation. The silencing of LINC01257 reduced tumor growth and had limited cytotoxicity [287].

Recently, Miao et al. used an LNP-encapsulated siRNA (LNP-siRNA) to silence Hsa_circ_0136666 in GC. Hsa_circ_0136666 competitively regulates PRKDC (a DNA-PK catalytic subunit) expression by sponging miR-375-3p. This results in the phosphorylation of PD-L1 (an immune checkpoint protein), which prevents its degradation. The phosphorylation of PD-L1 suppresses its immune function, thereby impairing the immune response to cancer. The use of the LNP-siRNA improved the efficacy of the anti-PDL1 drug and inhibited immune escape [288].

Epigallocatechin-3-gallate-lysozyme (EGCG-LYS) fibrils represent a novel siRNA delivery system for circMAP2K2 silencing, described by Dong et al. circMAP2K2 is abundantly expressed in GC, where it mediates the activation of the AKT/GSK3β/EMT signaling pathway. Furthermore, it enhances the proliferation and metastatic capacity of GC cells. The authors suggest that this novel delivery method has good circulatory stability, excellent biosafety, and in vivo anti-tumor capacity [289].

You et al. synthesized a novel siRNA delivery system, PEG-PCL (polycaprolactone)–PEI C14 (polyethyleneimine derivative)–SPION (PPPCS), based on superparamagnetic iron oxide nanoparticles (SPIONs). The intravenous injection of the PPPCS/siRNA complex silenced circ_0058051, resulting in the inhibition of tumor growth. Furthermore, the nanocomposite was nontoxic to the organs of nude mice [290].

## 6. piRNAs

### 6.1. Biogenesis of piRNAs

piRNA biogenesis is a complicated process, and many description models are based on *Drosophila* and mice, the best-characterized systems to date. Although some variation in piRNA biogenesis has emerged among the studied species, the process is generally conserved in its core components [291,292].

piRNAs are sncRNAs, approximately 24/25 to 31/32 nt in length, expressed mainly in the germ cells of animals. The majority of piRNA genes are organized into clusters at specific genomic loci. In these clusters, piRNAs align end to end or slightly overlap [293].

According to their genomic origin, piRNAs are divided into the following subclasses: transposon-derived [26], mRNA-derived [294], lncRNA-derived [294,295], snoRNA-derived [296,297], and processed transfer RNA (tRNA)-derived [32]. The piRNAs produced by TEs and repetitive sequences can be bidirectionally transcribed, generating sense and antisense piRNAs, with the latter being complementary to the DNA template. mRNA-derived piRNAs can be processed from full-length mRNAs [298] or from introns, exons, or the 3′ UTR of the pre-mRNA and recognize the same mRNA from which they originated [299,300,301].

piRNA biogenesis occurs in two phases, called the “primary” and “secondary” amplification cycles (also described as the “ping-pong cycle”) [302,303], which occur in somatic and germ cells or only in germ cells, respectively [304,305].

With primary amplification, the transcription of a piRNA cluster occurs by RNA polymerase II. The transcript formed is the piRNA precursor, which is a long ssRNA with a 5′ cap and a 3′ polyadenylated tail, which is then exported into the cytoplasm. There, secondary structures are resolved and the piRNA precursor is cleaved by MitoPLD and its co-factors into individual pre-piRNAs. The 5′ pre-piRNA is recognized and then loaded onto PIWI proteins, which are members of the highly conserved Argonaute protein family. After loading, further 3′ cleavage and concomitant methylation at the 3′ ends are required for the maturation of the piRNA molecule. In humans, mature piRNAs are expressed in germline and somatic tissues and generally have a uracil at the 5′ end, position +1, and an adenine at the +10 position, and they are 2′-O methylated at the 3′ end [299,306].

Once piRNA processing is completed, the mature piRNA–PIWI complex is formed and enters the nucleus, where it promotes TGS, or remains in the cytoplasm, where it can promote both PTGS and multiprotein interactions [307,308,309].

The secondary pathway is a mechanism that takes place in the cytoplasm and allows the rapid amplification of piRNAs. The antisense strands of piRNAs produced by primary amplification are bound by Aubergine (Aub) proteins; these are RNA-binding proteins that, in *Drosophila*, belong to the Piwi clade, formed by the Piwi, Aubergine, and Argonaute 3 (Ago3) proteins. The Piwi clade is part of the Argonaute protein family. In humans, the ortholog of Drosophila Aub is PIWIL1 [310].

The Aub–antisense-strand piRNA complex recognizes and cleaves the sense strand of a piRNA precursor. From this cleavage, a sense strand is generated, which is bound by Ago3. In contrast, Ago3 binds to sense-strand piRNAs and cleaves antisense piRNA precursors. In this case, an antisense piRNA is produced, which is loaded onto Aub proteins. However, Ago3 can only be loaded with secondary (Aub-generated) piRNAs, and the ping-pong cycle is initiated only by the piRNA–Aub complex. These piRNAs are then bound by PIWI proteins, forming the mature piRNA–PIWI complex [311,312,313].

### 6.2. Functional Role of piRNAs

piRNAs were first discovered in the testis of *Drosophila melanogaster* in 2001 [314] and were initially considered novel long siRNAs. Subsequently, piRNAs have been identified in about 44 species [315], including humans [316], for which there are more than 30,000 known piRNAs listed in the available piRNA-based databases. piRNAs show a tissue-specific expression profile, suggesting that they play important functional roles [317], but, for the most part, they are still unknown [318].

Functionally, piRNAs, like miRNAs, act through imperfect base pairing but, unlike the latter, piRNAs have considerable interspecific diversity and hence limited sequence conservation [303,319]. piRNAs are mainly involved in TGS and PTGS. Concerning TGS, the first reported function of piRNAs was the silencing of TE mobilization in fly germline cells [320], which is essential to maintain genome integrity. The inhibitory activity on TEs was later verified in other organisms, including humans [321]. Furthermore, it has been observed that piRNAs and Piwi proteins directly modify the chromatin structure and histone proteins in the nucleus by repressing the transcription of both TEs [322,323] and target genes [324].

In particular, piRNAs and Piwi proteins can repress transcription either by guiding DNMTs and promoting the methylation of CpG islands in promoter regions [324,325] or by interacting with the histone methylation machinery by regulating histone H3 lysine 9 (H3K9) [326] and lysine 4 (H3K4) [327,328] modification.

At the post-transcriptional level, piRNAs and Piwi proteins function similarly to miRNAs. The interaction with target molecules occurs by base pairing at the 5′ end of piRNAs and involves only a part of the piRNA sequence: 2–11 nt for strict base pairing and 12–21 nt for less strict base pairing [329]. piRNAs can bind to different RNA molecules, including transcribed pseudogenes [330], lncRNAs [331], and mRNAs [332]. The piRNA–mRNA interaction can occur by pairing in the 3′ UTR of mRNAs to promote deadenylation, with subsequent degradation via the mRNA decay machinery [291,332,333].

piRNAs are involved in post-transcriptional regulation also through the modulation of epigenetic m6A reversible modifications of RNAs [334]. Participating in this mechanism are “writer” methyltransferases, such as methyltransferase-like 3 (METTL3) and Wilms tumor 1-associated protein (WTAP), and “eraser” demethylases, such as AlkB homolog H5 (ALKBH5) and fat mass and obesity (FTO). The m6A modification of a mRNA affects the stability of the mRNA and regulates both the initiation and the elongation of its translation, but the precise fate of the mRNA depends on the functions of the different “readers” that bind the RNA molecule [335]. In general, the m6A modification can cause the destabilization of transcripts, which accelerates their degradation [336]. However, it has been described that the piRNA CHAPIR blocks the METTL3-mediated m6A methylation of Parp10 mRNA transcripts, which leads to an increase in the stability of the Parp10 mRNA, thus increasing in its expression [337]. Additionally, piRNA-30473 induces the upregulation of WTAP, which in turn increases the hexokinase 2 (HK2) m6A level, resulting in increased protein expression. Increased HK2 expression correlates with tumorigenesis in patients with diffuse large B-cell lymphoma (DLBCL) [338].

Finally, there are studies suggesting that piRNAs are also involved in the regulation of post-translational modifications such as phosphorylation [339,340].

A large body of literature suggests that piRNAs, as well as the associated Piwi pathway, may contribute to oncogenesis and tumor progression in various ways: (I) the anomalous expression of piRNAs is related to the development of different tumor hallmarks and to chemotherapy resistance [341,342,343,344,345,346,347,348]; (II) the deregulation of the piRNA–PIWI pathway can influence epigenetic mechanisms and lead to the altered regulation of gene expression with the consequent development of tumors [349,350,351,352]; and (III) impaired TE silencing may contribute to genomic instability, which is one of the hallmarks of cancer [353,354].

### 6.3. Therapeutic Applications of piRNAs

Numerous observations show that piRNAs’ abnormal expression is frequent in different types of cancer, but the exact mechanism behind their deregulation is still under investigation. To date, there are many preclinical studies on different tumors, including HCC [355,356,357], GC [358,359,360,361], CRC [362,363,364,365], osteosarcoma [366], LC [367,368,369,370], BrC [347,371,372,373], PrC [374,375], RCC [344,348,376], HNC [377], OC and CC [378,379], PaC [380], ALL [381], testicular cancer [382], ThC [383], and tongue squamous cell carcinoma [384].

These studies have explored piRNAs for their possible use as potential markers and provide new opportunities for cancer diagnosis, prognosis, or therapeutic approaches. Compared with other types of ncRNAs, piRNAs are a relatively new type of sncRNA, and aspects such as the complicated mechanisms through which they are generated, the difficulty in their identification, and the current lack of knowledge of their regulatory mechanisms represent limitations to their use in clinical practice. Furthermore, the role of piRNAs in the immune response and drug resistance in tumors is still largely unexplored. However, despite the many challenges in their clinical use, the interest in piRNAs and the availability of multiomics and sequencing technologies are leading to an improved understanding of their biological roles and their use as cancer therapy drugs.

### 6.4. piRNA-Based CTs

Research in this area is in its infancy; unfortunately, to date, there are no interventional CTs using piRNAs as therapeutic targets. However, several papers have suggested that piRNAs may be used as diagnostic or prognostic biomarkers in several types of cancer.

A recent study by Saha and collaborators suggests that human piR-23246, piR-32858, and piR-9137 may be used as biomarkers to diagnose PaC [385]. Xue et al. performed a meta-analysis on 27 studies to identify molecules in EV that could be used as non-invasive biomarkers for early GC diagnosis. From this study, several RNA molecules with diagnostic value emerge, including three piRNAs: piR-018569, piR-004918, piR-019308 [386]. Rui and coworkers identified five significantly upregulated exosome-derived piRNAs, piR-1029, piR-15254, piR-35395, piR-32132, and piR-43597, which could be used as biomarkers for HCC diagnosis [387]. Li et al. showed that serum exosomal piR-26925 and piR-5444 could be potential biomarkers for the diagnosis of LAC [388]. Nayak profiled miRNAs, piRNAs, and genes in GB U-87 MG cells and identified the targets related to progression and survival in GB patients. Among the identified targets, there are also some piRNAs that could be used as biomarkers in GB [389]. Peng found that the expression of piR-349843, piR-382289, piR-158533, and piR-002468 in urinary EV was significantly increased in PrC patients compared with a healthy control group, suggesting their use as diagnostic biomarkers [390]. Chang et al. observed that piR-13643 and piR-21238 were significantly upregulated in human PTC and suggested that they are promising novel biomarkers for the accurate detection of PTC [391]. Finally, Wang analyzed the serum of CRC patients and found that piRNAs piR-020619 and piR-020450 were upregulated compared to the controls. The authors indicate that the serum levels of the analyzed piRNAs show potential as specific early detection biomarkers for CRC [392].

In this context, there are only two observational CTs based on piRNA molecules: NCT06320418 and NCT04835454 (Table 3). NCT06320418 is a trial that started in 2022, and the current recruitment status is “active, not recruiting”. This trial aimed to investigate the regulatory role played by piR-823 in OC. Compared to non-tumor control tissues, piR-823 was deregulated in several cancers. Specifically, it was upregulated in HCC, CRC, and BC and downregulated in GC, showing that the mechanisms in which it is involved are tumor-specific. In fact, in HCC, it is involved in the pathophysiology of the tumor through the upregulation of the protein transforming growth factor b1 (TGF-b1) [355]. The TGF-b1 gene is frequently upregulated in tumor cells, and the protein regulates cell proliferation, differentiation, and growth [393]. In CRC, the upregulation of piR-823 promotes proliferation and inhibits apoptosis. Moreover, piR-823 upregulates heat shock transcription factor 1 (HSF1) expression by enhancing HSF1’s transcriptional activity through post-translational modification [340]. In BC, piR-823 promotes malignant cell proliferation, and its increased expression during cancer development may be associated with hormone levels [394]. In GC, piR-823’s downregulation is correlated with tumor growth inhibition [395]. However, the functional mechanisms of pir-823 in OC have not been investigated, so the above trial seeks to evaluate the possible use of pir-823 as a prognostic agent or as a potential target for drug development.

NCT04835454 is a trial that started in 2021, and the current recruitment status is not reported. The aim of the trial was to identify new biomarkers in PrC diagnosis. The potential biomarkers analyzed in CTs include different biological compounds, such as piRNAs, amino acids, and small nuclear RNAs. Currently, the early detection of PrC is based on two methodologies: digital rectal examination and the determination of the PSA levels in the blood. PSA testing seems not to be sufficiently specific for PrC as it often gives false positives [396], suggesting the need to identify new biomarkers.

## 7. circRNAs

### 7.1. Biogenesis of circRNAs

**Figure 5:**
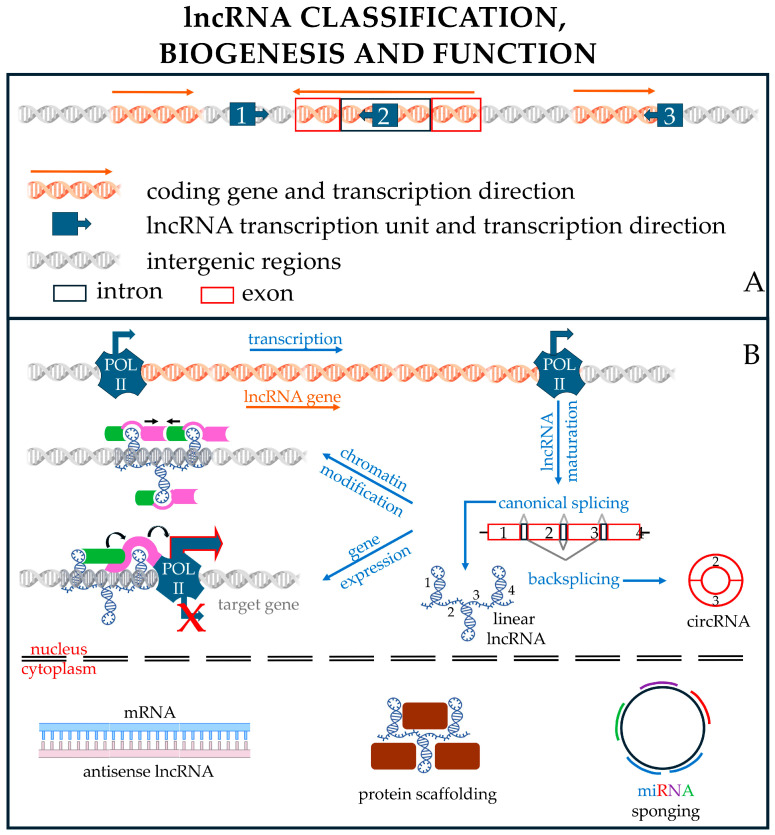
lncRNA biology. (**A**) Classification based on lncRNA gene position in the genome, which can be outside a coding region (intergenic lncRNA, sometimes named lincRNA) (1) or inside the intron of a coding gene (intragenic lncRNA) (2). Sometimes, the lncRNA is encoded on the complementary strand of a coding gene, resulting in an antisense lncRNA (3) with translation regulation functions. (**B**) Transcription and function of lncRNAs. Genes encoding lncRNAs are usually transcribed by Pol II and, in many cases, undergo maturation (i.e., capping, polyadenylation, and splicing) as their mRNA counterparts. Some transcripts can undergo a particular splicing mechanism called backsplicing, which generates circular RNAs (circRNA). lncRNAs may have roles either inside the nucleus or in the cytoplasm. In the figure, some examples of these roles are reported. Inside the nucleus, the lncRNA can modify the chromatin structure (e.g., modifying nucleosome positioning to achieve more compact chromatin, black arrows), or it can recruit proteins (green and pink elements), which can alter the gene expression profile (e.g., transcription factors or methylases), possibly causing chromatin modification and either enhancing (Pol II, top) or repressing (Pol II bottom, with the red X indicating the inhibition of transcription) target gene expression. Curved arrows indicate that the green protein interacting with the lncRNA can recruit the pink protein, which, in turn, interacts with Pol II to modify target gene expression. In the cytoplasm, lncRNAs (either linear or circular) may interact with target mRNAs (antisense) or with proteins (scaffolds) or may sponge microRNAs (either different or multiple copies of the same miRNA). Image partially built using freely available resources at NIH BioArt (https://bioart.niaid.nih.gov/).

circRNAs are a type of ncRNA consisting of a ssRNA that forms a covalently closed circular structure between the 3′ and 5′ ends of the strand. Depending on the genomic origin, circRNAs can consist of a single exon or multiple contiguous or non-contiguous exons [397], from truncated forms of exons [398], from introns only [399], or from a combination of exons and introns [400,401] (Figure 5). Some circRNAs can originate from mitochondrial RNAs or from intron self-splicing that occurs during the maturation process of some constitutive ncRNAs, such as snRNAs, rRNAs, and tRNAs [27,28,29,30,31].

The biogenesis of a circRNA starts in the cell nucleus from a precursor (pre-mRNA) of protein-coding genes synthesized by RNA polymerase II (RNA Pol II), which are spliced using non-canonical mechanisms such as backsplicing and its variants, including lariat-driven circularization, intron-pairing-driven circularization, and RBP-driven circularization [402].

The backsplicing mechanism is regulated by both cis-regulatory elements (e.g., splice sites, enhancers, silencers, inverted Alu repeats) [403] and trans-acting factors (e.g., spliceosome factors, RNA helicases, and RNA-binding proteins) [404]. In this type of splicing, the 3′ splice site of the exon of a pre-mRNA is joined to the 5′ splice site of an upstream exon of the same mRNA molecule. Backsplicing allows the head-to-tail closure of a molecule because a downstream splice donor (5′ splice site) joins backwards to an upstream splice acceptor (3′ splice site).

The canonical splicing of pre-mRNAs usually removes introns between adjacent exons. However, sometimes, as in exon skipping, introns are removed from the pre-mRNA along with the exons, forming a structure called a lariat. The lariat can be further spliced to form a circRNA from either the exon alone or the intron alone [405,406]. In the first case, the circRNA is formed through a phosphodiester bond between the 3′ hydroxyl of the 3′ exon and the 5′ phosphate of the 5′ exon [407], while, in the second case, the circRNA is formed through a phosphodiester bond between the 2′ hydroxyl of the 5′ intron and the 5′ phosphate of the 3′-intron [399].

Intron pairing is a mechanism that occurs through the complementary pairing of flanking introns on both sides of the exons. The pairing of introns allows the formation of a ds stem. At the end of the stem, on one side, there is a downstream splice donor site, and, on the other, there is the upstream splice acceptor site of the pre-circRNA. The proximity of these sites favors reverse splicing events that remove the intron stem, allowing the joining of the sites (donors and acceptor) and the circularization of the RNA. The intron-pairing mechanism is strictly dependent on the pairing ability of complementary intron sequences (CIS), which are often derived from TEs that are inverted repeats, such as Alu elements [408,409]. The deletion of CIS from endogenous gene loci alters the formation of circRNAs [410,411], while some chromosomal translocations present in cancer cells create new CIS, promoting the generation of new circRNAs [412,413].

RBPs represent a category of proteins whose elements act as trans-acting factors in the regulation of circRNAs’ biogenesis [414]. Using high-throughput screening, approximately 1500 RBPs have been identified in the human genome, of which approximately 9% are likely involved in carcinogenesis [415,416,417]. There are two main types of RBPs, dsRBPs and ssRBPs. The former have dsRNA-binding domains and can regulate circRNA formation by affecting the RNA-pairing stability. ssRBPs do not have dsRNA-binding domains, recognize specific motifs present in the intronic sequence, and can dimerize with each other. RBPs promote contact between donor and acceptor splice sites. However, there are also RBPs that inhibit the formation of circRNAs, such as adenosine deaminase RNA-specific 1 (ADAR1). ADAR1 binds dsRNA to mediate adenosine-to-inosine (A-to-I) RNA editing, which is a post-transcriptional modification of the RNA sequence. ADAR1-guided editing modifies the circRNA precursor sequence, thereby altering the base pairing between complementary sequences in flanking introns, resulting in the negative regulation of circRNA biogenesis. Conversely, it has been reported that ADAR1 depletion can upregulate the formation of circRNAs [418,419]. In general, circRNA biogenesis is a very complex process, and one aspect that further complicates the understanding of this mechanism is that individual gene loci can generate multiple circRNAs (alternative circularization) [400]. This is possible by using different splice sites to form the backsplicing junction (a process called an alternative backsplicing event) and by using various types of alternative splicing [397,420].

The nucleotide sequences of many circRNAs can be post-transcriptionally modified, influencing different biological aspects of the molecule, such as its half-life, translation, nucleocytoplasmic export, and localization. The most commonly observed modifications are A-to-I editing and the m6A modification [421,422]. The m6A modification is particularly frequent, and its effect depends on “reader” proteins that recognize the modification sites [422].

Mature circRNAs can have different fates: they can carry out their activity in the nucleus, they can localize in the cytoplasm or in organelles such as mitochondria [30,423] or ribosomes [424], or they can be loaded into exosomes—thus acting outside the cells from which they originate. The nucleus contains mainly exon–intron or intron-only circRNAs. The mechanisms by which circRNAs are retained in the nucleus have not been fully elucidated. The cytoplasmic transport mechanism may depend on several adaptor proteins and some nuclear exportins. Adaptor proteins bind the circRNA molecule in relation to the length of the molecule. Huang and collaborators demonstrated that, in human cells, circRNAs of different sizes are transported into the cytoplasm by binding to different proteins, such as UAP56 and URH49, which regulate the nucleo-cytoplasmic transport of long (>1298 nt) and short (<356 nt) circRNAs, respectively [425]. However, other factors may be related to cytoplasmic transport, such as m6A modifications [426] or RNA duplex structures within circRNAs [427]. In both cases, however, the involved molecular mechanisms are not yet characterized. The circRNA-bound adaptor proteins are then exported into the cytoplasm through the involvement of nuclear exportins such XPO4 [428] or XPO2 [429]. Exosome RNA sorting involves specialized RNA sequences and/or secondary structures associated with RBPs. The precise mechanism by which circRNAs are sorted into exosomes remains undetermined [430,431].

Studies demonstrating that some circRNAs contain open reading frames that could be translated into small peptides are continually increasing. However, since circRNAs possess neither a 5′-cap nor a 3′-poly(A) tail, they adopt strategies that are different from canonical cap-dependent translation, such as the use of IRES plus additional regulatory sequence elements [432], mechanisms associated with A-to-I RNA editing [433] or m6A modification [434,435,436,437,438], the use of an exon junction complex [439,440,441], and rolling circle translation [442,443]. Many of the peptides produced by these mechanisms are implicated in carcinogenesis, suggesting their potential use as therapeutic targets.

### 7.2. Functional Role of circRNAs

The history of circRNA discovery began in 1976 with the description of a pathogenic viroid containing a covalently closed ssRNA [444]. Then, at the beginning of the 1990s, the pioneering works of Nigro et al. [445], Cocquerelle et al. [446], and Capel et al. [447] were published, which demonstrated the presence of circRNAs also in eukaryotes, including humans. However, an increase in circRNA research occurred in only around 2010, thanks to improvements in RNA-seq technologies, together with the development of specialized computational pipelines. Nowadays, it is estimated that human circRNAs amount to over 100,000. This is an impressive estimation considering that the circular nature of the molecule and the low abundance of some circRNAs often make their identification challenging. However, over the years, in addition to methods such as Northern blotting or RT-qPCR, high-sensitivity and -throughput strategies have been developed, such as rolling circle amplification [448,449], RNA sequencing (RNA-seq) coupled with NanoString technologies [450], microarrays [451], fluorescence in situ hybridization [452], and RNA-seq [453].

The analysis of circRNAs has also allowed us to improve our knowledge of the structural elements related to the functions that characterize the molecules. In general, a circRNA sequence may possess one or more of the following structural elements: short, inverted repeats and hairpin structures, MREs, or IRES.

The presence of inverted repeats and hairpin structures confers protein-binding properties to circRNAs. Protein interaction has several biological implications, including protein stabilization [454], degradation [455], localization [456,457], translation regulation [458,459], or scaffold functions [460,461].

Some circRNAs possess MRE sites that allow competitive binding to miRNAs. This binding inhibits the repressive function of miRNAs and consequently allows the expression of miRNA targets. This mechanism is called RNA sponging. A typical miRNA sponge consists of multiple miRNA-binding sites, each containing mismatches at intermediate positions to prevent the activation of the endoribonuclease function of Ago2 [462]. circRNAs can contain either multiple binding sites for the same miRNA or multiple miRNA-binding sites for different miRNAs. For example, circZNF91 contains 24 miR-23b-3p target sites [463], while circCCDC66, whose elevated expression in CRC is associated with poor prognosis, possesses multiple miRNA-binding sites, including those for miR-33b, miR-93, and miR-185 [464].

There are numerous examples in the literature implicating the deregulation of the miRNA sponge function of circRNAs in the development of numerous pathologies, including cancer. In this context, circRNAs can either repress or promote oncogenesis, and, in general, the alteration of the circRNA–miRNA–mRNA axis (composed of ceRNAs) influences every aspect of carcinogenesis, including the response to treatment [465,466,467,468,469,470].

CircRNAs, in addition to their function as miRNA sponges to regulate the activity of miRNAs, also have additional roles, including miRNA storage (e.g., CDR1-AS) [471] and transport (e.g., circDlc1(2)) [472]. The cerebellar degeneration-related 1 (CDR1) gene expresses an antisense circular transcript, known as CDR1-AS, which interacts with several miRNAs, including miRNA-671 and miRNA-7. CDR1-AS binds miRNA-7, for which it has 70 MREs, and transports it to a specific site. Subsequently, binding to miRNA-671 induces the degradation of the circRNA and the release of miRNA-7. The regulation of this mechanism is not fully understood but it has been hypothesized that it could depend on spatial–temporal signals [473]. circDlc1(2) binds some mRNAs associated with glutamate receptor signaling (gluRNAs) and allows the correct localization of miR-130b-5p at synaptic regions, where a gluRNA is localized. An interesting aspect of this circRNA is that the binding to the miRNA does not induce its inhibition. In fact, the control of the subcellular localization of the miRNA promotes its activity, suggesting a new mechanism compared to the canonical one of miRNA inhibition [474].

Nuclear circRNAs may be involved in the regulation of gene expression by modulating the activity of RNA polymerase II, epigenetic mechanisms, mRNA splicing, and rRNA processing. Some circRNAs, such as circEIF3J and circPAIP2, have been described to bind U1 spliceosomal RNA through an RNA–RNA interaction. U1 spliceosomal RNA is the snRNA component of U1 snRNP, a complex involved in the assembly of the spliceosome. The circRNA–U1 snRNP interaction could regulate the Pol II transcription complex at the promoters (cis via) of parental genes to enhance gene expression [418]. Furthermore, some circRNAs, such as circFECR1, can activate the transcription of their parental genes through the modulation of epigenetic mechanisms. circFECR1 is an FLI1 exonic circRNA, and, by inducing DNA hypomethylation in the CpG islands of the FLI1 promoter, it enhances its transcription. In addition, in BrC, FLI1 regulates the formation of metastases through the epigenetic regulation promoted by its exonic circRNA [475]. Other nuclear circRNAs, such as circ-MBL [476] and circSMARCA5 [477], regulate mRNA splicing. circ-MBL works through competitive binding with the splicing machinery, while circSMARCA5 has binding motifs for several RBPs, including the splicing factors SRSF1, SRSF3, and PTB2. circANRIL, through interaction with nucleolar protein 14 (NOP14), blocks pre-rRNA processing and ribosome biosynthesis, inducing cell apoptosis [478].

Finally, some circRNAs, through binding to specific proteins, such as transcription factors, allow their nuclear translocation. For example, the interaction of circ-Amotl1 with c-myc not only allows the protection of c-myc from degradation but also the translocation of the protein into the nucleus, where it can perform its function [479].

Currently, numerous manuscripts have highlighted that the alteration of circRNA pathways is responsible for the development of many pathologies, including cancer. Unfortunately, the majority of the works on circRNAs focus on the consequences of the alteration of the expression levels of a specific circRNA and not on the causes. However, despite the few available studies, it is possible to describe some of the factors that lead to the deregulation of circRNAs.

Some alterations could depend on genomic mutations that directly affect intronic flanking regions, leading to the generation of new oncogenic circRNAs. An example is the SLC34A2–ROS1 (solute carrier family 34 member 2 and ROS proto-oncogene 1) gene fusion that results from a chromosomal translocation, creating a new flanking complementary sequence with canonical splicing sites. This involves the biogenesis of two new circRNAs: F-circSR1 and F-circSR2. Both promote cell migration in NSCLC and are important for cancer progression [412]. circMLL is also a byproduct of a chromosomal translocation and promotes transcriptional pausing, proteasome inhibition, chromatin reorganization, and DNA breakage in the early stages of the development of acute leukemias [480].

Qiu and collaborators, through the screening of circRNA expression in relation to CNV in LAC, identified circPRKCI. CNV represents one of the main causes of structural variation in the genome, involving both duplications and deletions of chromosome fragments, and is involved in tumorigenesis. circPRKCI is a proto-oncogenic circRNA derived from the LAC 3q26.2 amplicon. circPRKCI is overexpressed in tumors and promotes cell growth and migration by functioning as a sponge for both miR-545 and miR-589 [481].

Altered epigenetic mechanisms can influence the biogenesis of circRNAs. For example, some circRNAs are transcriptionally silenced through hypermethylation or altered histone modifications at their host gene promoters, leading to a subsequent reduction in circRNA expression [482,483,484].

Alterations in splicing factors or the production of abnormally alternative splicing isoforms is a condition often detected in tumors [485,486]. Kong et al. report that, in PDAC, the upregulation of circARFGEF2 is dependent on the KRAS^G12D^ mutation. This mutation is responsible for the overexpression of the RNA splicing factor Quaking (QKI)-5, which, in turn, facilitates cir-cARFGEF2 biogenesis by binding the QKI-binding motifs and reverse complement sequence in introns 3 and 6 of the ARFGEF2 pre-mRNA. Thus, the overexpression of circ-ARFGEF2 induces lymph node metastasis in PDAC [487,488]. The biogenesis of a circRNA depends on the correct execution of the backsplicing mechanisms and its variants, which is guaranteed by the action of cis-acting elements and trans-acting splice factors. Thus, alterations at the level of cis- and trans-elements can influence circRNA biogenesis [489]. Fernandez and collaborators performed circRNA-seq on lymphoid and myeloid cell lines expressing the most common splicing factor (SF) mutations, showing the general upregulation of circRNAs. In addition, each mutant SF is characterized by its own set of upregulated circRNAs [490]. The RNA-binding protein QKI is involved in alternative splicing and binds QKI-binding motifs present on some introns, contributing to the biogenesis of circRNAs. Conn and collaborators demonstrated that, during EMT in immortalized human mammary epithelial (HMLE) cells, hundreds of circRNAs are upregulated in a QKI-dependent manner [491]. Additionally, the depletion or inhibition of core spliceosome components, including the U1 and U2 snRNPs, results in circRNA upregulation [404,492].

The stabilization of the molecule by some post-transcriptional modifications, such as m6A, could also contribute to the accumulation of the circRNA, as observed for circRPS6KC1 in PrC [493].

Finally, some circRNAs can be loaded into exosomes. The incorrect loading of circRNAs could have a dual effect: on the one hand, the cell will experience a decrease in its circRNA load; on the other hand, exosomes lead to the enrichment of incorrect circRNAs in inappropriate locations.

Mechanistically, the altered expression level of circRNAs, both endogenous and exosomal, observed in cancer cells has been associated with the deregulation of key cellular signaling pathways, such as PI3K/AKT/mTOR, Wnt, notch and hippo, p53/Bcl-2, and TGF-β/Smad, which in turn, in the context of carcinogenesis, influences cell proliferation, EMT, invasion, metastasis, apoptosis, angiogenesis, and the pharmacological response [494,495].

In recent years, the study of circRNAs has produced a large amount of data, which has made it necessary to organize them into databases. There are different types of databases that facilitate study and consultation for those working in the field. For example, MiOncoCirc provides information about the associations between circRNAs and cancer [496], and Lnc2Cancer 3.0 contains not only circRNA–cancer associations but also information on the regulatory mechanisms, biological functions, and clinical applications of circRNAs in cancer [497]. CircFunBase [498], deepBase [499], and circBank [500] are three additional examples of databases that collect data on the interactions of circRNAs with RNAs and proteins. Finally, the circVAR database collects SNPs and small insertions and deletions (INDELs) in putative circRNA regions. The use of circRNA variants in GWAS allows the identification of many cancer-based somatic variants, suggesting novel mechanisms for cancer development [501].

### 7.3. Therapeutic Applications of circRNAs

Therapeutic applications are mainly limited by the poor characterization of the biological functions performed by circRNAs. However, the understanding of RNAs’ structure and function and advances in nucleic acid synthesis/modification technology have led to the development of synthetic circRNAs that could be used as therapeutic agents [502,503]. This approach enables the synthesis of circRNAs and protein expression at multiple levels, from the specific cellular compartment or within cells and tissues, with the aim of pursuing targeted therapy. For the therapeutic use of synthetic circRNAs, some technical aspects must be optimized. In fact, the synthesis of this molecule can lead to the incorporation of exogenous noncoding sequences or to a final product that is not sufficiently purified and can activate a patient’s immune response after administration. In this context, the choice of the vector to be used must also be made with particular care to avoid adverse responses. Currently, the delivery vectors can be broadly categorized into viral (adenoviral, retroviral, and AAV vectors) and nonviral vectors (physical methods, chemical methods, and biologically derived vectors), with the latter being preferred [502] (Figure 3).

Regarding the expression of circRNAs in disease, upregulation is more common than downregulation. This aspect has led to the use of different oligonucleotide-based strategies that are designed to achieve circRNA knockdown by targeting the backsplicing junction site, allowing one to restore circRNA expression to a healthy level. These strategies include the use of siRNA [504], shRNA [505], ASO [506], or CRISPR/Cas systems [506], which represent a powerful gene editing tool that specifically knocks down a circRNA without interfering with its homologous mRNA [424].

### 7.4. CircRNA-Based CTs

The use of circRNAs in clinical practice is currently limited to their use as biological markers for early diagnosis. For example, PrC diagnosis was significantly improved in clinical studies using the combination of circRNA detection and PSA testing [507]. The use of circRNAs as biological markers depends on some particularly advantageous characteristics that they possess. The closed structure of a circRNA ensures greater stability than linear RNAs. This feature depends on the ability to resist exonuclease-based degradation and allows circRNAs to have a longer half-life (ranging from 19 h to 24 h) than linear transcripts with identical nucleotide sequences (ranging from 4 h to 7 h) [508].

circRNAs present in exosomes are also protected from degradation, thus allowing them to exert their action far from the cell of origin. Furthermore, exosomes are not only easily detectable but also present in many human biofluids, such as urine, saliva, gastric juice, cerebrospinal fluid, ascites, and blood, and could also be used for liquid biopsies [495], allowing for fast and minimally invasive detection procedures.

circRNA-based therapies are currently limited to preclinical studies only. At present, a search on the ClinicalTrials.gov website produces only 10 CTs based on circRNAs: nine observational trials evaluating the use of circRNAs as markers (Table 4) and one interventional trial (phase 1) evaluating the use of circFAM53B-219aa DC in cancer vaccine monotherapy.

The trial NCT06649253 started in 2025, and the current status is “not yet recruiting”. The aim of this CT was to analyze the network of lncRNA/circRNA/miRNA/mRNAs involved in the regulation of CD9 in B-ALL. B-ALL is the most common cancer in children and is characterized by a rather high percentage of patients with relapse. CD9 is a transmembrane protein associated with B-ALL relapse [509,510], and understanding the mechanisms that regulate its expression could be useful to identify relapse markers.

NCT06617585 started in 2024, and the current status is “not yet recruiting”. The aim of the trial was to evaluate the possible diagnostic role of circDENND4C [511] in OC.

The trial NCT06042842 started in 2023, and the current status is “not yet recruiting”. This trial is evaluating hsa_circ_0004001 [512] as a diagnostic biomarker for HCC.

The trial NCT05934045 started in 2023, and the current status is “active, not recruiting”. This trial aims to evaluate the possibility of using circRNAs as circulating prognostic and/or predictive biomarkers in ALCL, an aggressive T-cell pediatric lymphoma.

The trial NCT05771337 started in 2023, and the current status is “not yet recruiting”. The aim of this trial is to evaluate hsa_circ_0001785 (Circ-ELP3) and hsa_circ_100219 (Circ-FAF1) [513] in serum samples from BrC patients as possible diagnostic and prognostic biomarkers.

The trial NCT05377736 started in 2022, and the current status is “enrolling by invitation”. This pilot study aims to describe the distribution of molecular alterations in benign and malignant thyroid nodules at the DNA and RNA levels, including circRNAs, in the Danish population.

The trial NCT04464122 started in 2020, and the current status is “recruiting”. The goal of this study is to identify novel biomarkers, including circRNAs, from tumor-educated platelets (TEPs) in the diagnosis and assessment of the treatment response in pulmonary and gastro-entero-pancreatic NENs. Platelets are involved in the processes of tumorigenesis; during their life cycle, in the bloodstream, they absorb and enrich substances produced by the tumor—hence, they are named TEPs [514]. NENs are a group of heterogeneous tumors with neuroendocrine differentiation that can arise from cells distributed within the neuroendocrine system [515].

The trial NCT04584996 started in 2020, and the current status is “unknown”, meaning that the trial has passed its completion date and the status has not been verified in the last 2 years. Studies with an unknown status are considered closed studies. In this trial, the aim was to define the circRNA expression profile in PDAC and to find, among the dysregulated ones, a candidate for a new, clinically relevant diagnostic or prognostic biomarker.

The trial NCT03334708 started in 2017, and the current status is “recruiting”. In this trial, blood samples are being analyzed for molecules, such as specific circRNAs, that could be used as biomarkers to diagnose PaC in the early stages of the disease and monitor the response to treatment.

The trial NCT06530082 started in 2024, and the current status is “not yet recruiting”. This trial is phase 1 and is aimed at evaluating the safety, efficacy, and tolerability of circFAM53B-219aa DC [516] vaccine monotherapy and its combination with camrelizumab in the treatment of HER2-negative advanced BrC.

## 8. lncRNAs

### 8.1. Biogenesis of lncRNAs

lncRNAs are a heterogeneous class of RNA molecules longer than 200 nucleotides that do not encode proteins. In the last few decades, they have emerged as key regulators of gene expression and play significant roles in various biological processes and diseases, including cancer. Their biosynthesis involves transcription by RNA polymerase II, similar to what happens to mRNAs. Likewise, their maturation follows comparable steps, including capping at the 5′ end, splicing, and polyadenylation at the 3′ end. However, unlike mRNAs, lncRNAs often exhibit lower expression levels and higher tissue specificity [65,517]. The transcription of lncRNAs is tightly regulated by various transcription factors and epigenetic modifications, which ensure their precise expression in different cellular environments (Figure 5).

### 8.2. Functional Role of lncRNAs

lncRNAs regulate gene expression at multiple levels, including chromatin remodeling, transcriptional control, and post-transcriptional processing [518]. They can interact with DNA, RNA, and proteins to modulate various cellular processes, such as cell cycle progression, apoptosis, and differentiation [519]. Some lncRNAs act as molecular scaffolds, bringing together different proteins to form functional complexes [520], while others serve as decoys to sequester regulatory molecules [517]. Furthermore, lncRNAs can influence mRNA stability and translation by binding to complementary sequences or interacting with RNA-binding proteins [521]. They also play roles in the formation of nuclear bodies and the maintenance of the nuclear architecture [522]. lncRNAs have gained attention for their potential as biomarkers in cancer diagnosis and prognosis due to their specific expression patterns in different cancer types. For instance, the lncRNA PCA3 is used as a biomarker for PrC [471]. lncRNAs can be detected also in body fluids, making them suitable for non-invasive diagnostic tests [472,523,524,525]. Additionally, they are involved in cancer progression by regulating oncogenes and tumor suppressor genes, thus representing potential therapeutic targets [526].

### 8.3. Therapeutic Applications of lncRNAs

Therapeutic strategies targeting lncRNAs include antisense oligonucleotides, siRNAs, and small molecules designed to modulate their function. Recent studies have shown that lncRNAs can also mediate the resistance to chemotherapy and radiotherapy, highlighting their importance in personalized cancer treatment.

Pseudogenes are a particular class of lncRNAs that resemble functional genes. They arise through gene duplication or retrotransposition but accumulate mutations that prevent them from encoding functional proteins. Despite being considered “junk DNA”, some pseudogenes are transcribed into RNAs and can regulate gene expression by acting as decoys for miRNAs or by generating regulatory RNAs. Pseudogenes can also contribute to genomic instability and cancer development by serving as sources of genetic variation [527,528].

Thus, lncRNAs and pseudogenes represent significant components of the noncoding genome with crucial roles in gene regulation and disease. Their unique properties and functions offer promising avenues for the development of novel diagnostic and therapeutic strategies in cancer.

### 8.4. lncRNA-Based CTs

There is excellent potential for the development of cancer therapies targeting lncRNAs, but, currently, these are only in the early stages of development. Instead, CTs evaluating their use as diagnostic, prognostic, or predictive markers (detection of relapse, response to treatment, monitoring of therapeutic efficacy) are more numerous (Table 4).

Unfortunately, the results of the completed CTs have not yet been published. However, from the CTs presented in Table 4, two interesting observations emerge. The first is that approximately 46% of the trials (12 out of 26 CTs) have started in the last three years, suggesting a greater interest in experimentation; the second is that the most frequently evaluated lncRNAs in CTs are lnc-GC1 (NCT05397548, NCT05647941, NCT05334849), H19 (NCT05943093, NCT04767750), and HOTTIP (NCT06544005, NCT04729855), which, taken together, constitute about 27% (7 out of 26 CTs) of the listed trials. These lncRNAs are studied in GC (lnc-GC1), in HCC (H19, HOTTIP), in ALL (H19), and in CRC (HOTTIP), and, according to data communicated for the year 2022 by the GCO (a platform curated by the WHO and the IARC), they are among the tumors with the highest incidence and mortality in the world.

Sun et al. demonstrated that GC-associated long noncoding RNA1 (lncRNA-GC1) is upregulated in GC, where it influences several aspects of carcinogenesis, such as the tumor size, metastasis, and prognosis. The authors suggest that, mechanistically, lncRNA-GC1 acts as a scaffold for two protein factors involved in histone modifications: WDR5 (a histone methyltransferase) and KAT2A (a histone acetyltransferase). The action of lncRNA-GC1 allows the correct localization of the two proteins, thus ensuring their normal function on the target genes. The abnormal expression of lncRNA-GC1 affects the functions of WDR5 and KAT2A, inducing altered gene expression that leads to the formation of GC [529]. Subsequent studies have identified lncRNA-GC1 as a highly expressed GC-specific lncRNA in both GC cells and exosomes. In particular, the work of Guo et al. demonstrated a correlation between lncRNA-GC1 levels and the GC stage [530]. This implies that, in GC patients, the detection of circulating exosomal lncRNA-GC1 provides clinically important diagnostic and prognostic information [531].

H19 is a lncRNA involved in many regulatory cellular functions [532], and its altered expression has been observed in many tumors [533]. Depending on the type analyzed, H19 can behave as an oncogene or a tumor suppressor [534]. For example, in ALL (both B-ALL and T-ALL) [535] and HCC [536], H19 behaves as an oncogene. Specifically, in ALL, the observed expression level of H19 was significantly higher than in controls [535], and it was correlated with an unfavorable prognosis [537]. Zhao et al. suggest a mechanism in which increased H19 could competitively bind miR-19a and miR-19b, resulting in the upregulation of inhibitor of DNA binding 2 (ID2), which is an important regulator in cell proliferation and differentiation [538,539]. In HCC, H19 is involved in carcinogenesis and the recurrence, metastasis, and chemoresistance of tumors. In addition, it was observed that H19 expression increased in the liver cancer stem cells, tissue, and plasma of patients with HCC, while it decreased after a partial/complete therapeutic response [536]. Although the biological function of H19 in HCC remains to be elucidated, Nokkeaw et al. suggest that H19 may act as a molecular sponge for miR-107 to promote cyclin-dependent kinase 6 (CDK6) expression and cell cycle progression; this axis may explain the correlation between H19 overexpression and increased cell proliferation in HCC [540].

The lncRNA HOTTIP (homeobox A (HOXA) transcribed at the distal tip) is encoded by a genomic region at the 5′ tip of the HOXA locus, which plays a key role in embryologic development. HOTTIP binds the histone methyltransferase complex, allowing its correct localization to specific genomic loci, including the HOXA gene cluster. HOTTIP is an example of the cis regulation of gene expression by a lncRNA, since its action allows the trimethylation of histone H3 at lysine 4 (H3K4me3) and the activation of HOXA genes [541]. HOTTIP expression levels are altered in many tumors [542,543,544], including HCC [545] and CRC [546]. In HCC patients, HOTTIP upregulation is correlated with advanced tumor stages and a poorer prognosis. The mechanisms by which HOTTIP is involved in HCC carcinogenesis are multiple. For example, Wang and coworkers suggest that HOTTIP promotes the activation of HOXA genes, which, in turn, is correlated with cell proliferation, migration, and invasion [541,545]. Wei et al. indicate that HOTTIP promotes HCC by regulating glutamine metabolism [547]. The regulation of glutamine metabolism is a type of metabolic reprogramming that is considered a hallmark of cancer cells [548]. In the literature, there are several works that exploit the properties of HOTTIP, such as its stability in serum and the high expression levels in HCC patients compared to healthy controls, to evaluate a possible role as a tumor marker. In this context, Bao et al. and Kim et al. indicate HOTTIP as a potential marker for the early diagnosis of HCC [549,550]; in addition, Bao and coauthors correlate high serum HOTTIP expression levels with increased metastasis formation in HCC patients [550]. HOTTIP is also upregulated in human primary CRC tissues, where it promotes cell proliferation, migration, and invasion [546]. Several preclinical studies have shown that HOTTIP can be used as a possible marker for the early diagnosis of CRC [551], as a drug response prediction marker [552], as a predictive marker of risk, and as a prognostic marker [553].

## 9. The Role of Mitochondrial ncRNAs in Cancer

Despite its small genome size and limited protein production capacity, the mitochondrion has a very complex ncRNA profile. Indeed, like its nuclear counterpart, mtDNA encodes several ncRNA species (mt-ncRNAs), including piRNAs, miRNAs, and sncRNAs derived from lncRNAs or from the processing of tRNA and rRNA genes. Increasing evidence suggests that mt-ncRNAs play important roles in cellular homeostasis, and their alteration induces mitochondrial dysfunction associated with the development of several diseases, including cancer. Although the discovery of mt-ncRNAs is quite recent and their biological functions are poorly understood [554], the importance of some of them is emerging since they modulate processes such as cell division, apoptosis, cell proliferation, and metastasis formation [555].

### 9.1. SncmtRNA, ASncmtRNA-1, and ASncmtRNA-2

Three lncRNAs originate from the mitochondrial rRNA 16S (*mt-RNR2*) gene: a mt-ncRNA (SncmtRNA) and two antisense transcripts, ASncmtRNA-1 and ASncmtRNA-2. SncmtRNA is expressed in proliferating and normal tumor cells but not in non-proliferating control cells, suggesting a role in cell division. ASncmtRNA-1 and ASncmtRNA-2 are expressed in normal proliferating human cells, are downregulated in tumor tissue, and are not expressed in non-dividing cells [556,557]. The expression pattern of ASncmtRNAs suggests two important considerations: (I) they may act as tumor suppressors and be involved in malignant cell transformation processes; (II) the different expression profile observed allows us to specifically distinguish between normal and tumoral cells, and this aspect can be used in a therapeutic strategy.

### 9.2. LIPCAR

Long intergenic noncoding RNA predicting cardiac remodeling (LIPCAR) is a chimeric mitochondrial transcript in which the 5′ half maps to the antisense strand of the mitochondrial gene lncCyt b and the 3′ half maps to the antisense strand of the mitochondrial gene COX2. Although the literature on LIPCAR is mainly focused on its role in cardiac diseases, its involvement in cell proliferation and carcinogenesis has been recently explored in the process of phenotypic switching. This process allows cells to change their phenotype to a “highly synthetic phenotype”, which in turn permits cells to acquire various characteristics, such as proliferative and migratory abilities, increased protein synthesis, and the secretion of collagen, elastin, and matrix metalloproteinases [558]. Alterations in the switching process have been observed in cancer and influence malignant transformation by promoting invasion, metastasis, and tumor growth [559,560].

LIPCAR is upregulated in both HCC patients and the HCC HepG2 cell line. LIPCAR’s overexpression in HepG2 induces the inhibition of apoptosis and increases cell proliferation, migration, and invasiveness through increased levels of N-cadherin, Vimentin, and Claudin and decreased levels of E-cadherin. Furthermore, BALB/c nude mice injected with LIPCAR-overexpressing HepG2 cells formed HCC tumors and showed greater metastasis. In this system, LIPCAR overexpression promotes tumor growth and metastasis via the activation of the EMT process [561].

Finally, a positive correlation between LIPCAR and the TGF-β/Smad pathway in atrial muscle tissues was demonstrated [562]. This pathway is involved in cell growth, differentiation, motility, and apoptosis, and its alteration has been associated with both the development and progression of many tumors, as well as with drug resistance. Thus, it is plausible to speculate that the mechanisms of action that correlate LIPCAR with the TGF-β/Smad pathway may also function in carcinogenesis; however, further studies are needed to support this hypothesis.

### 9.3. lncCytB and mcPGK1

The mitochondrial antisense strand of the CytB gene transcribes two lncRNAs called lncCytB and mitochondrial circRNA for translocating phosphoglycerate kinase 1 (mcPGK1). Studies comparing the localization of lncCytB in HL7702 (normal hepatic cells) and in HepG2 (hepatoma cells) show that, in HL7702, lncCytB is primarily localized in the mitochondria, while, in HepG2, it is still localized in the mitochondrion but mainly in the nucleus. Although the nuclear function has not been fully clarified, the authors suggest the aberrant shuttling of lncCytB in the HepG2 nucleus, where it may function in the epigenetic regulation of genes involved in carcinogenesis [563]. Zhang et al., using both mouse models and human cell lines, such as AC16 (human cardiomyocytes), HL-1 (mouse cardiac muscle cells), and neonatal mouse cardiomyocytes (NMCM), suggest that lncCytB could function as a ceRNA in the lncCytB/miR-103-3p/PTEN/AKT axis. This mechanism is particularly interesting for several reasons: (I) the role of miR-103-3p in carcinogenesis has been described in various tumors [564,565]; (II) in NSCLC, the miR-103-3p/PTEN interaction regulates AKT-inducing cell proliferation and invasion [566]; (III) miR-103-3p sponging by lncRNAs is involved in the regulation of different hallmarks of cancer [567,568]. Although yet to be demonstrated, these considerations suggest that the lncCytB/miR-103-3p/PTEN/AKT axis may be involved in carcinogenesis.

In another report, Chen et al. separated liver tumor-initiating cells (TICs) from non-TICs from primary liver cancer and analyzed the circRNAs differentially expressed between the two cell types. Circular mcPGK1 was more enriched in TICs than in non-TICs, and subsequent investigations suggested its involvement in the molecular circuit, which inhibits OXPHOS and promotes the Warburg effect [569].

### 9.4. MDL1 and MDL1AS

The mitochondrial D-loop encodes two lncRNAs called mitochondrial D-loop 1 (MDL1), which originates from the sense strand, and mitochondrial D-loop 1 antisense (MDL1AS), which originates from the antisense strand.

An analysis of A549 LC cells reveals that MDL1 participates in retrograde signaling to mediate the regulatory control of nuclear genes involved in cell cycle regulation. In particular, MDL1 mediates the formation of a ternary complex, MDL1/p53/Tid1, that inhibits p53 nuclear translocation [570]. Protein p53 is a transcription factor with hundreds of targets; therefore, the inhibition of p53 nuclear translocation has a broad regulatory effect on the expression of nuclear genes and influences the expression of numerous genes involved in apoptosis, cell cycle arrest, autophagy, metabolism, DNA repair, and feedback mechanisms [571].

Garrido et al. analyzed the expression levels of MDL1 and MDL1AS in different tumor tissues (colon, rectum, breast, and larynx cancer) and compared them to those in normal surrounding tissues. The authors found no differences in the MDL1 levels between tumor and control samples, while the MDL1AS levels were variable and tumor-type-dependent. In fact, the MDL1AS levels were lower in CRC and higher in BrC and LarC. The authors explored the role of MDL1AS using ds interfering RNA (DsiRNA) to downregulate MDL1AS in colon (HCT-116) and breast (MCF7 and MDA-MB-231) cancer cell lines. The results showed that, in HCT-116 cells, the downregulation of MDL1AS reduced both cell growth and migration, while the opposite behavior was observed in MDA-MB-231 cells. A molecular analysis was subsequently performed using both markers of apoptosis (BAD, BAX, BCL2) and the cell cycle (CDK4, CDKN1A, CCNA1). The results showed that BCL2, CDK4, CDKN1A, and CCNA1 were downregulated in both cell lines upon MDL1AS downregulation, compared to control cells not treated with DsiRNA. In contrast, BAD and BAX showed downregulation only in HTC-116 and MDA-MB-231, respectively, when compared to control cells not treated with DsiRNA [572].

### 9.5. circ-COX2

circ-COX2 is a new circRNA generated by backsplicing from the transcription of the mitochondrial COX2 gene. An analysis of exosomes isolated from the plasma of patients with CLL showed the enrichment of circ-COX2 compared to normal controls, suggesting a role of circ-COX2 in CLL. A functional analysis performed on different CLL cell lines showed that the downregulation of circ-COX2 affects mitochondrial function and induces both the suppression of cell proliferation and increased apoptosis, while the upregulation of circ-COX2 is correlated with disease progression and reduced survival [573].

### 9.6. circ-ND5 and ND6

Liu and coworkers identified two circRNAs, circ-ND1 and circ-ND5, that were upregulated in HCC compared to controls, i.e., normal adjacent cancer tissues. circ-ND1 and circ-ND5 are transcribed from the mitochondrial ND1 and ND5 genes, respectively, and appear to be involved in facilitating mitochondrial protein import. However, their potential role in HCC pathogenesis needs further exploration [574].

### 9.7. Mitochondrial ncRNA-Based Therapies in CTs

Due to their central role in many cellular processes, mitochondria are suitable therapeutic targets for the treatment of various diseases, including cancer. Molecules designed to inhibit proteins/enzymes required for their function are often tested [575,576], as well as those that target mtDNA replication, which has a critical role in the formation of new mitochondria [577]. The molecules evaluated in most CTs target protein/enzyme components of mitochondria; however, to date, there are at least two CTs that have investigated mt-ncRNAs, namely ASncmtRNA-1 and ASncmtRNA-2, as therapeutic targets.

ASncmtRNAs were chosen in several preclinical studies that have highlighted two key factors for this purpose. First, the analysis of the expression pattern of ASncmtRNAs allows us to specifically distinguish between normal and tumoral cells. This implies that a therapeutic agent targeting ASncmtRNAs could preferentially reach abnormal cells, thus increasing the specificity of the treatment and decreasing its side effects. Second, several studies have used an ASO, namely Andes-1537, complementary to the loop region of both ASncmtRNAs. The use of this construct has contributed to understanding the biological roles of both ASncmtRNAs and, consequently, their potential as oncological therapeutic targets. Using cells derived from primary and metastatic ccRCC and an orthotopic xenograft model of ccRCC, both treated with Andes-1537, an increase in apoptosis and a decrease in proliferative capacity and metastasis formation were generally observed. At the molecular level, ASncmtRNAs knockdown induces a reduction in cellular proliferation through the reduction of cyclins B1 and D1 and the inhibition of metastasis formation through the reduction of N-cadherin [578]. The works of Fitzpatrick [579] and Bendek [580] show that the knockdown of ASncmtRNAs influences the function of some key factors required for cell cycle progression and ensures genomic integrity. In particular, they showed the downregulation of CDK1, CDK4, survivin, aurora kinase A (AURKA), and topoisomerase IIα (TOPO2A). In addition, they also demonstrated that Andes-1537 induced the upregulation of both mitochondrial encoded hsa-miR-4485-3p and hsa-miR-1973, together with nuclear hsa-miR-5096 and hsa-miR-3609. The miRNA hsa-miR-4485-3p induces the downregulation of cyclins B1 and D1, while hsa-miR-1973 seems to have no effect on the regulation of cyclins. Instead, an analysis of nuclear hsa-miR-5096 and hsa-miR-3609 using the TargetScanHuman prediction tool identified the canonical binding sites on the CDK1 mRNA [579].

Preclinical studies led to the design of a phase I CT (NCT02508441). This study was completed in 2018, with the aim to determine the safety, tolerability, and MTD of Andes-1537. Twenty-two patients with advanced unresectable solid tumors participated in this study. The results were encouraging because the authors not only demonstrated the safety of the drug and established the MTD (600 mg injected subcutaneously every two weeks), but also showed that the treatment with Andes-1537 stabilized the disease progression beyond 6 months for one patient with PaC and one patient with ChC [227]. Andes-1537 was also tested in an additional CT (NCT03985072), which started in 2019 (Table 2). In this CT, 67 patients were enrolled, with different advanced solid tumors, such as GBTC, CC, GC, PaC, and CRC. Although completed in 2022, the results of this trial have not yet been published.

## 10. Conclusions

In recent years, the interest in ncRNAs has grown significantly, and this has contributed to a better understanding of the complex cellular mechanisms in which they are involved and their importance in ensuring cellular homeostasis. In fact, in every analyzed tumor, it is possible to find an altered ncRNA expression pattern. The alteration of this pattern depends mainly on ncRNAs of nuclear origin, but, recently, a role has been highlighted also for ncRNAs of mitochondrial origin. In fact, despite the small size of the mitochondrial genome compared to its nuclear counterpart, this organelle can transcribe an intricate and varied network of ncRNAs. Although several aspects of mitochondrial function in cancer are still far from being elucidated, the study of mtDNA-encoded ncRNAs could further expand the list of possible anti-cancer targets. In this context, it will be interesting to continue the evaluation in CTs of Andes-1537, which is the only ASO directed against a mt-ncRNA.

The role that nuclear and mitochondrial ncRNAs play in oncogenesis and in the various aspects that lead to tumor progression and development suggests huge potential for the development of cancer therapies targeting ncRNAs, but these are currently only in the early development phase. There are still many challenges to overcome for their use in clinical practice. One of these concerns their functional characterization, which is very fragmented. To date, there is an evident imbalance between the number of various classes of ncRNAs annotated in their specific databases, all of which are constantly updated, and the number of ncRNAs analyzed at the bench. In addition, ncRNAs often have multiple cellular targets that include RNAs, DNA, and proteins, forming intricate interaction networks that are not known in detail. Even the genetic/molecular mechanisms that regulate ncRNAs’ biogenesis and cellular functions are often poorly understood. With the current knowledge, the use of ncRNAs as therapeutic targets could contribute to several side effects, such as off-target deregulation, the alteration of healthy cellular molecular pathways, toxicity, and adverse immune responses. In addition to the issues related to the functional knowledge of ncRNAs, there are further challenges to overcome, such as efficient and targeted delivery systems that, in addition to ensuring the stability, bioavailability, and correct dosage at the cellular level of the ncRNA, allow the delivery of the molecules specifically to tumor cells. Finally, the half-lives of these drugs and their vectors also need to be strongly regulated to avoid their depletion in target cells and ensure their correct excretion after exerting the desired effect. Although the therapeutic use of ncRNAs is at the early stages, these molecules are also being investigated as exciting biomarkers in different CTs for their diagnostic, prognostic, and predictive potential, as well as their therapeutic efficacy or for the monitoring of the pathology over time. These new biomarkers could improve the current oncological clinical protocols, with a significant effect in terms of improving patients’ prognosis, thanks, for example, to early diagnosis, as well as contributing to the choice of the best therapy to be performed. As such, in the near future, they will likely be included in panels for the molecular characterization of patients and their diseases. Overall, despite the challenges, ncRNAs are increasingly emerging as promising tools for a new era of precision and personalized medicine in cancer therapy.

## Figures and Tables

**Table 1:** This table lists all abbreviations used in this review. The first and second columns contain the abbreviations and corresponding definitions of the general terms mentioned in the text. The third and fourth columns show the abbreviations and corresponding definitions of the different types of cancer mentioned in the text.

Abbreviation General Term	Definition	Abbreviation Cancer	Definition
AAV	Adeno-associated virus	ALCL	Anaplastic large-cell lymphoma
agshRNA	Ago2-dependent shRNA	ATLL	Adult T-cell leukemia/lymphoma
ASO	Antisense oligonucleotide	ALL	Acute lymphoblastic leukemia
ceRNA	Competing endogenous RNA	B-ALL	B-type acute lymphoblastic leukemia
CNV	Copy number variation	BlC	Bladder cancer
CT	Clinical trial	BrC	Breast cancer
DNMT	DNA methyltransferase	CC	Cervical cancer
ds	Double-stranded	ccRCC	Clear cell renal cell carcinoma
EBV	Epstein–Barr virus	CLL	Chronic lymphocytic leukemia
EMT	Epithelial–mesenchymal transition	ChC	Cholangiocarcinoma
eRNA	Enhancer RNA	CRC	Colorectal cancer
EV	Extracellular vesicles	DLBCL	Diffuse large B-cell lymphoma
GCO	Global Cancer Observatory	EOC	Epithelial ovarian cancer
GWAS	Genome-wide association studies	GB	Glioblastoma
HTLV-1	Human T-cell leukemia virus type 1	GC	Gastric cancer
IARC	International Agency for Research on Cancer	GcC	Gastric cardia cancer
IRES	Internal ribosome entry site	HCC	Hepatocellular carcinoma
lincRNA	Long intergenic ncRNA	HNC	Head and neck cancer
LNA	Locked nucleic acid		
LNP	Lipid nanoparticle	LAC	Lung adenocarcinoma
miRNA/miR	microRNA	LarC	Laryngeal cancer
MRE	miRNA recognition element	LC	Lung cancer
mRNA	Messenger RNA	Me	Melanome
MTD	Maximum tolerated dose	MF-CTCL	Mycosis fungoides-type cutaneous T-cell lymphoma
mtDNA	Mitochondrial DNA	MPM	Malignant pleural mesothelioma
mt-ncRNA	Mitochondrial noncoding RNA	NEN	Neuroendocrine neoplasm
m6A	N6-methyladenosine	NSCLC	Non-small-cell lung cancer
NamiRNA	Nuclear activating miRNA	OC	Ovarian cancer
NCI	National Cancer Institute	PaC	Pancreatic cancer
nDNA	Nuclear DNA	PDAC	Pancreaticobiliary cancer
NER	Nucleotide base repair	PM	Peritoneal mesothelioma
nPC	Non-protein-coding	PTC	Papillary thyroid carcinoma
nt	Nucleotide	PrC	Prostate cancer
PEG	Polyethylene glycol	RC	Renal cancer
PC	Protein-coding	Sa	Sarcoma
piRNA	Piwi-interacting RNA	SCLC	Small-cell lung cancer
PS	Nucleotide phosphorothioate	TNBC	Triple-negative breast cancer
PSA	Prostate-specific antigen	ThC	Thyroid cancer
PTGS	Post-transcriptional gene silencing		
RBP	RNA-binding protein		
RNAi	RNA interference		
rRNA	Ribosomal RNA		
shRNA	Short hairpin RNA		
siLNA	Combination of mixmers and siRNA		
sncRNA	Short noncoding RNA		
snoRNA	Small nucleolar RNA		
SNP	Single-nucleotide polymorphism		
snRNA	Small nuclear RNA		
snRNP	Small nuclear ribonucleoprotein		
SNV	Single-nucleotide variant		
ss	Single-stranded		
TE	Transposable element		
TERC	Telomerase RNA component		
TGS	Transcriptional gene silencing		
tRNA	Transfer RNA		
UTR	Untranslated region		
VM	Vasculogenic mimicry		
WHO	World Health Organization		

**Table 2:** List of main oncology CTs, described in detail in the text, which use ncRNAs as therapeutic targets. Data retrieved from ClinicalTrials.gov website on 22 December 2024.

Drug Strategy ^1^	Drug Name	Target	Cancer Type ^2^	Recruitment Status ^3^	Clinical Trial Phase	Clinical Trial Identifier ^4^	Ref. ^5^
LNA	LNA-i-Mir-221	miRNA-221	BrC, CRC, GC, GB, HCC, OC, PC, PM	Completed	Phase 1	NCT04811898	[222]
	Cobomarsen/ MRG-106	miR-155	MF-CTCL, CLL, DLBCL, ATLL	Completed	Phase 1	NCT02580552	[223]
			MF-CTCL	Terminated	Phase 2	NCT03837457	n/a
			MF-CTCL	Terminated	Phase 2	NCT03713320	n/a
miRNA-M	MRX34	miR-34a	HCC, Mel, SCLC, TNBC, Sa, BlC, RC, OC	Terminated	Phase 1	NCT01829971	[224]
	MRX34	miR-34a	Me	Withdrawn	Phase 1	NCT02862145	n/a
	Targomir	miR-16	MPM, NSCLC	Completed	Phase 1	NCT02369198	[225]
	INT-1B3	miR-193a-3p	Adv. mal.	Terminated	Phase 1	NCT04675996	[226]
ASO	Andes-1537	ASncmtRNA-1 ASncmtRNA-2	AUST	Terminated	Phase 1	NCT02508441	[227]
			GBTC, CC, GC, PaC, CRC	Completed	Phase 1	NCT03985072	n/a

^1^ Drug strategy represents the molecule used as a therapeutic agent. LNA, locked nucleic acid; miRNA-M, miRNA mimic; ASO, antisense oligonucleotide. ^2^ For human cancer abbreviations, please refer to Table 1. ^3^ indicates the current recruitment status. Completed: the study was concluded normally, and the participants are no longer undergoing visits or treatment; terminated: the study was stopped prematurely and will not resume, and the participants are no longer receiving any visits or treatment; withdrawn: the study was stopped before enrolling the first participants. Status descriptions are in accordance with the definitions provided by ClinicalTrials.gov. ^4^ CT identifier is the identification code given to each clinical study upon registration on the ClinicalTrials.gov website. ^5^ n/a: no specific reference is available for the cited CT.

**Table 3:** Summary of oncology CTs evaluating sncRNAs as biomarkers. Data retrieved from ClinicalTrials.gov website on 22 December 2024.

ncRNA Type	Clinical Trials Identifier ^1^	Start Year	Recruitment Status ^2^	Biomarker Purpose	Sample Analyzed	Cancer Type ^3^
miRNA	NCT06738225	2025	Not yet recruiting	Diagnostic	Serum	CRC
miRNA	NCT06610851	2024	Recruiting	Diagnostic	Blood	Gliomas, grades 2 and 3
miRNA	NCT06203496	2024	Recruiting	Diagnostic	Blood	Gliomas, grade 4
miRNA	NCT06730035	2024	Active, not recruiting	Prognostic	Blood	CRC
miRNA	NCT06702891	2024	Not yet recruiting	Diagnostic	Multiple biological samples, e.g., serum and tissue	GcC
miRNA	NCT06224166	2023	Recruiting	Diagnostic	Tissue samples, blood and saliva evaluated	HNC
miRNA	NCT06001099	2023	Recruiting	Diagnostic	Blood	Gynecologic cancers
miRNA	NCT05901376	2023	Recruiting	Diagnostic	Blood	GA
miRNA	NCT06240195	2023	Recruiting	Prognostic	Blood, plasma	TNBC, metastatic phase
miRNA	NCT05697224	2023	Not yet recruiting	Diagnostic and prognostic	Urine	Bilharzial BlC
miRNA	NCT05746858	2023	Not yet recruiting	Prognostic	Plasma, serum	DLBCL, relapsed/ refractory
miRNA	NCT06320184	2023	Active, not recruiting	Diagnostic	Blood	LC
piRNA	NCT06320418	2022	Active, not recruiting	Prognostic	Tissue	OC
piRNA	NCT04835454	2021	Unknown status	Diagnostic	Not specified	PrC

^1^ Clinical Trials Identifier is an identification code given to each clinical study upon registration at the ClinicalTrials.gov website. ^2^ indicates the current recruitment status. Not yet recruiting: the study has not started recruiting participants; recruiting: the study is currently recruiting participants; active, not recruiting: the study is ongoing and participants are receiving an intervention or being tested, but potential participants are not currently being recruited or enrolled; unknown: a study whose last known status is known (recruiting, not yet recruiting, active not recruiting) but it has passed its completion date and its status has not been verified within the last 2 years; enrolling by invitation: the study selects its participants from a population, or group of people, decided in advance by the researchers—therefore, these studies are not open to all who meet the eligibility criteria, but only to people specifically invited to participate. Status descriptions are in accordance with the definitions provided on the ClinicalTrials.gov website. ^3^ For human cancer abbreviations, please refer to Table 1.

**Table 4:** Summary of oncology CTs evaluating lncRNAs as biomarkers.

ncRNA Type	Clinical Trials Identifier ^1^	Start Year	Recruitment Status ^2^	Biomarker Purpose	Samples Analyzed	Cancer Type ^3^
circRNA	NCT06649253	2025	Not yet recruiting	Diagnostic and prognostic	Bone tissue and blood	B-ALL
circRNA	NCT06617585	2024	Not yet recruiting	Diagnostic	Serum	EOC
circRNA	NCT06042842	2023	Not yet recruiting	Diagnostic	Plasma	HCC
circRNA	NCT05934045	2023	Active, not recruiting	Prognostic	Serum	ALCL
circRNA	NCT05771337	2023	Not yet recruiting	Diagnostic	Serum	BrC
circRNA	NCT05377736	2022	Enrolling by invitation	Diagnostic	Tissue and blood	ThC
circRNA	NCT04464122	2020	Recruiting	Diagnostic and prognostic	Blood	NENs (pulmonary and gastro-entero-pancreatic)
circRNA	NCT04584996	2020	Unknown	Diagnostic and prognostic	Tissue, blood, bile, and biopsy	PDAC
circRNA	NCT03334708	2017	Recruiting	Diagnostic	Blood	PDAC
lncRNA	NCT06531850	2024	Recruiting	Diagnostic	Serum	LC
lncRNA	NCT06307249	2023	Recruiting	Prognostic and predictive	Blood, tissue	CRC, LC, OC, BrC
lncRNA	NCT06334835	2023	Recruiting	Diagnostic and prognostic	Bone marrow mononuclear cells	T-ALL
lncRNA	NCT06065592	2019	Recruiting	Prognostic and predictive	Blood and tissue	CRC
lncRNA	NCT06544005	2022	Active, not recruiting	Prognostic	Blood	HCC, metastatic phase
lncRNA	NCT04729855	2022	Active, not recruiting	Prognostic and diagnostic	Blood and tissue	CRC
lncRNA	NCT05943093	2023	Not yet recruiting	Predictive	Unspecified	ALL
lncRNA	NCT05270174	2023	Not yet recruiting	Diagnostic	Urinary exosomes	BlC
lncRNA	NCT05397548	2022	Unknown	Diagnostic	Blood exosomes and tissue	GC
lncRNA	NCT05088811	2021	Unknown	Diagnostic	Serum	HCC
lncRNA	NCT04269746	2020	Unknown	Diagnostic	Blood	CRC
lncRNA	NCT03469544	2018	Unknown	Diagnostic	Blood	ThC
lncRNA	NCT03738319	2018	Unknown	Diagnostic and prognostic	Blood exosomes	HG-SOC
lncRNA	NCT05647941	2018	Unknown	Predictive	Circulating exosomes	GC
lncRNA	NCT03057171	2015	Unknown	Predictive	Tissue	GC
lncRNA	NCT06534242	2022	Completed	Prognostic and predictive	Blood	CRC
lncRNA	NCT05708209	2022	Completed	Diagnostic	Saliva	OSCC
lncRNA	NCT05141383	2022	Completed	Diagnostic and prognostic	Blood and urine	PrC
lncRNA	NCT06357689	2021	Completed	Predictive	Blood	BrC
lncRNA	NCT06432413	2021	Completed	Prognostic	Serum	CRC
lncRNA	NCT04767750	2020	Completed	Predictive	Blood	HCC
lncRNA	NCT06531902	2020	Completed	Diagnostic	Blood	CRC
lncRNA	NCT06427278	2019	Completed	Predictive	Blood	CRC
lncRNA	NCT05334849	2018	Completed	Predictive	Blood	GC
lncRNA	NCT05730855	2022	Completed	Diagnostic	Saliva	OrC
lncRNA	NCT03830619	2017	Completed	Diagnostic	Serum exosomes	LC

^1^ CT Identifier is an identification code given to each clinical study upon registration at the ClinicalTrials.gov website. ^2^ indicates the current recruitment status. Recruiting: the study is currently recruiting participants; active not recruiting: the study is ongoing and participants are receiving an intervention or being tested, but potential participants are not currently being recruited or enrolled; not yet recruiting: the study has not started recruiting participants; unknown: a study whose last known status is known (recruiting, not yet recruiting, active not recruiting) but it has passed its completion date and its status has not been verified within the last 2 years; completed: the study concluded normally, and the participants are no longer undergoing visits or treatment. Status descriptions are in accordance with the definitions provided by the ClinicalTrials.gov website. ^3^ For human cancer abbreviations, please refer to Table 1.
